# ANKRD22, a novel tumor microenvironment-induced mitochondrial protein promotes metabolic reprogramming of colorectal cancer cells

**DOI:** 10.7150/thno.37472

**Published:** 2020-01-01

**Authors:** Tianhui Pan, Jingwen Liu, Song Xu, Qiao Yu, Hongping Wang, Hongxiang Sun, Jia Wu, Yue Zhu, Jianwei Zhou, Yongliang Zhu

**Affiliations:** 1 Laboratory of Gastroenterology Department, Second Affiliated Hospital of Zhejiang University School of Medicine, Hangzhou, Zhejiang 310009, China;; 2 Gynecology Department, Second Affiliated Hospital of Zhejiang University School of Medicine, Hangzhou, Zhejiang 310009, China;; 3 Laboratory of Natural Drug, College of Animal Sciences, Zhejiang University, Hangzhou, 310058, China;; 4 Key Laboratory of Tumor Microenviroment and Immune Therapy of Zhejiang Province, Hangzhou, Zhejiang 310009, China.; 5 College of Stormotologry, Wenzhou Medical University, Zhejiang325035, China.

**Keywords:** Colorectal cancer, Tumor microenvironment, Cancer-initiating cells, ANKRD22, Metabolic reprogramming

## Abstract

**Background:** The leading cause of poor prognosis in colorectal cancer (CRC) is the presence of colorectal cancer-initiating cells (CCICs). The interplay between the tumor microenvironment (TME) and CRC cells induces reacquisition of initiating cell characteristics, but the underlying mechanisms remain elusive.

**Methods:** Candidate molecules were screened by global differential cDNA expression profiles of CCICs, which were enriched from patient-derived tumor xenograft models. Luciferase reporters and chromatin immunoprecipitation assays were used to explore the mechanism of TME factors regulating the transcription of *ANKRD22*. The effects of Ankyrin repeat domain-containing protein 22 (ANKRD22) on energy metabolism were monitored by extracellular flux and ^13^C-based metabolic flux analysis. Mass spectrometry was used to identify the interacting partners of ANKRD22. Morphological changes of CCICs overexpressing ANKRD22 were observed by electron microscopy. The effects of ANKRD22 on mitochondrial lipid metabolism were analyzed by lipidomics.

**Results:** We identified a novel nucleus-encoded mitochondrial membrane protein, ANKRD22, which was upregulated in CCICs. We found that ANKRD22 was induced by the p38/MAX pathway activated by different TME stimuli. As a key transcription factor, MAX promoted the transcription of *ANKRD22*. Expression of ANKRD22 promoted glycolysis associated with a decrease in ATP/ADP and an increase in AMP/ATP levels, which were related to its interaction with pyruvate dehydrogenase kinase isoform 1 (PDK1) and multiple subunits of ATP synthase. Further, in CCICs, ANKRD22 cooperated with the lipid transport protein, Extended Synaptotagmin-1 (E-Syt1), to transport excess lipids into mitochondria and reduced the number of mitochondria in an autophagy-independent manner, thus meeting the metabolic requirements of CCICs.

**Conclusion:** ANKRD22 induced by TME promotes the metabolic reprogramming of CRC cells. Our study has identified ANKRD22/E-Syt1 as a potential target for eradicating CCICs.

## Introduction

Colorectal cancer (CRC) is one of the most common malignant tumors 1. Metastasis and recurrence of CRC are closely related to the presence of colorectal cancer-initiating cells (CCICs) in CRC tissues. CCICs are a small subpopulation of CRC cells, which possess self-renewal and strong tumorigenic properties, are resistant to chemoradiotherapy, and contribute to the poor prognosis of CRC 2, 3. In addition to hereditary mutations in normal colorectal stem cells, recent studies suggest that the primary source of CCICs is the reacquisition of cancer-initiating cell (CIC) characteristics by the reprogramming of non-CCICs. The tumor microenvironment (TME) plays a vital role in this non-CCICs-to-CCICs conversion 4-6.

The TME is hypoxic, nutrient deficient, infiltrated by inflammatory cells (e.g., macrophages and lymphocytes), and enriched with cytokines and senescent tumor cells. Many studies have demonstrated the influence of TME on the ability of non-CICs to reacquire CIC characteristics through different pathways. It has also been shown that hypoxia can activate HIF-1α and increase the expression of induced pluripotent stem cell reprogramming factors 7. TGF-β increases ZEB1 transcription by switching the ZEB1 promoter from a bivalent state to an active state. TGF-β also enhances epithelial-mesenchymal transition (EMT), which promotes the reprogramming of breast cancer cells 8. CXCL-8 promotes EMT and the stemness of CRC cells through the Akt-Slug pathway 9. IL-22 promotes the activities of STAT3 and the H3K79 methyltransferase DOT1L, which induce the stemness-associated transcriptional factors NANOG, SOX2, and POU5F1 in CRC cells 10. IL-17 secreted by regulatory T cells (Tregs) can promote the expression of DCLK1, POU2F3, ALDH1A1, and IL17RC in pancreatic cancer cells and increase their CIC characteristics 11. Senescent tumor cells can also reacquire stemness through p16^INK4a^, p21^CIP1^, p53, and H3K9me3. Recent studies have revealed that senescent B-lymphoma cells generated by chemotherapeutic agents can regain CIC characteristics by activating the Wnt pathway 12, 13.

Cell reprogramming includes metabolic and nuclear reprogramming, both of which are involved in cell fate. Metabolic reprogramming, essential for the survival and growth of CICs, adapts metabolic levels needed to meet the metabolic needs of initiating cells and affects nuclear reprogramming through transcriptional or epigenetic regulation 14-16. Mitochondria are the central organelle where metabolic reprogramming occurs in tumor cells. In the early stage of reprogramming of tumor cells, glucose metabolism transitions from oxidative phosphorylation (OXPHOS) to aerobic glycolysis 17. Recent evidence that enhanced lipid metabolism during reprogramming may perform another important function has attracted considerable interest 18, 19. Mitochondrial fission is likely to be a part of lipid metabolism reprogramming 20. Several studies have reported a smaller number, lower activity, and less immature morphology of mitochondria in stem cells 21, 22. The dynamic balance (e.g., through continuous fusion and fission) during bi-directional interconversions between CIC and non-CIC states is a significant concept that requires further study 23. However, the key molecules and mechanisms of mitochondrial metabolic remodeling induced by the TME leading to non-CCIC-to-CCIC conversion remain elusive.

In this study, we identify a novel mitochondrial membrane protein, Ankyrin repeat domain-containing protein 22 (ANKRD22), which is induced by the TME through p38 pathway and involved in the metabolic reprogramming of CRC cells, mainly through promoting glycolysis. We show that in CCICs, ANKRD22 coordinates with the lipid transport protein, Extended Synaptotagmin-1 (E-Syt1), to transport excess lipids into mitochondria and reduce the number of mitochondria in an autophagy-independent manner.

## Materials and Methods

### Cells and cell culture

Cells were cultured in the following media (Corning, USA) supplemented with 10% fetal bovine serum (Corning, USA) and 100 μg/ml of gentamicin (Sangon Biotech, China) at 37 °C in a humidified 5% CO_2_ atmosphere: CRC SW620 and SW480 cells, lung adenocarcinoma NCI- H1299 cells and cervical cancer HeLa cells in 1640 medium; CRC HT-29 cells in McCoy's 5A medium; RKO cells in MEM; Ls 174T cells and human embryonic kidney 293T cells in DMEM; T84 cells, primary CRC P1, and P2 cells (established in our laboratory from primary colorectal cancer tissues) in DMEM/F12 medium. Except for primary CRC, all cells above were purchased from the Cell Bank of Shanghai Branch of the Chinese Academy of Sciences (Shanghai, China). Different conditions were applied to cells for mimicking TME, such as hypoxia, which was created with GENbag (Biomerieux, France); glucose deprivation (glucose-free culture medium); glucose-free medium supplemented with 3 mM glucose was used as low-glucose condition; ROS induction (100, 200, and 500 μM H_2_O_2_); senescence induction (5 μg/ml CPT-11); cytokine stimulation (5 ng/ml TGF-β or 30 ng/ml IFN-γ, Sinobiological, China).

### Western blotting (WB)

Cell lysis was performed as per our previously described procedure 24. Briefly, cells were harvested and incubated in RIPA lysis buffer (50 mM Tris-HCl buffer, pH7.4, 150 mM NaCl, 1 mM EDTA, 1% NP-40, 0.25% deoxycholate, 1× protease inhibitor cocktail (Calbiochem, USA)) for 30 min. The supernatant was collected by centrifugation at 13,000 rpm for 15 min. Protein lysates were subjected to 12% sodium dodecyl sulfate-polyacrylamide gel electrophoresis (SDS-PAGE). Then the proteins were transferred to nitrocellulose membranes (Sartorius Stedim, Germany). The membranes were washed with pH 7.5, 50mM Tris-HCl buffer saline containing 0.05% Tween-20 (TBST) and subsequently blocked with 5% non-fat milk TBST at room temperature (RT) for 1 h and incubated with the primary antibody at 4 °C overnight with gentle shaking. The following primary antibodies were used in this study: β-Tubulin (HuaBio, China), p38 MAPK (Cell Signaling Technology, USA), phos-p38 MAPK (Thr180/Tyr182) (Cell Signaling Technology), phos-SAPK/JNK (Thr183/Tyr185) (Cell Signaling Technology), MAX (Abcam, UK), VDAC1 (Abcam), MDH2 (Abcam), Histone H3 (Cell Signaling Technology), AMPK (Cell Signaling Technology), phos-AMPK(Thr172) (Cell Signaling Technology), phos-ACC (Ser79) (Cell Signaling Technology), PDK1 (HuaBio), ANT2 (Celling Signaling Technology), Flag Tag (HuaBio), LC3B (Cell Signaling Technology), SQSTM1/P62 (Cell Signaling Technology), E-Syt1 (Proteintech, China), DAG (LifeSpan BioSciences, USA), PIP2 (Invitrogen, USA), p53 (Cell Signaling Technology). Subsequently, the membranes were washed 5 times with TBST and incubated with HRP-labeled goat anti-rabbit IgG (H+L) antibody or HRP-labeled goat anti-mouse IgG (H+L) antibody (1:3000, Jackson Immunoresearch Laboratories, USA) at RT for 1 h. After washing with TBST 5 times, the membranes were incubated in ECL substrate solution (Perkin Elmer, USA) for 1 min and imaged on C-DiGit® Blot Scanner (LI-COR® Biosciences, USA).

### Organoid culture

1×10^5^ cells were trypsinized to single cells, resuspended with 1 ml of serum-free DMEM, mixed with 1 ml of growth factor-reduced Matrigel matrix (Corning, USA) and seeded into 6-well ultra-low attachment plates (Corning, USA). After keeping at 37 °C for 1.5 h, serum-free medium (DMEM/F12 medium with 1× B27, 20 μg/L EGF, 20 μg/L b-FGF, 4 mg/L insulin, 0.4% BSA and 100 μg/ml gentamicin) was added. Cells were cultured at 37 °C in a humidified 5% CO_2_ atmosphere for 5-7 days, and then the medium was removed and replaced by cell recovery solution (Corning, USA) mixed with collected Matrigel matrix at the ratio of 1:1. After gently shaking at 4 °C for 2 h, the mixture was centrifuged at 1000 rpm for 10 min and cell precipitate was harvested for subsequent experiments. For testing the organoid formation ability, 100 cells were counted, resuspended with 100 μl of serum-free DMEM medium, mixed with 100 μl of growth factor-reduced Matrigel matrix, and seeded along the edge of the well in a 24-well plate. The number of organoids was counted under the microscope after 7 days.

### Establishment of *Ankrd22^-/-^* C57BL/6 mice

The production of *Ankrd22*^-/-^ C57BL/6 mice using CRISPR/Cas9 technology was commissioned to Cyagen Biosciences (China). Two pairs of primers were used to test the knockout of *Ankrd22*. Pair 1: 5'-GCTGCCCTAAAGTCTTTCCTTCC-3' (Forward), 5'-CATAGCTCATTATAGTTGCACATTCTTTT G-3' (Reverse), fragment size of 635bp, annealing temperature of 57 °C; Pair 2: 5'-GCTGCCCTAAAGTCTTTCCTTCC-3' (Forward), 5'-GGGAGTATCGCCATTGAAGCTATCT-3' (Reverse), fragment size of 383 bp, annealing temperature of 57 °C. The amplified products were purified for DNA sequencing analysis. Homozygous *Ankrd22^-/-^* mice were used for subsequent breeding and experiments.

### Oxygen consumption rate (OCR) and extracellular acidification rate (ECAR)

Seahorse XF96 Extracellular Flux Analyzer (Seahorse Bioscience, USA) was used to detect cellular OCR and ECAR. On the first day, experimental and control cells were seeded into Seahorse XF96 cell culture microplates (Seahorse Bioscience, USA), and the XFe96 sensor cartridges (Seahorse Bioscience, USA) were hydrated. At least 5 replicates were performed for the measurement of each group. On the following day, for OCR detection, microplates were incubated with basic culture medium (17 mM glucose, 1 mM sodium pyruvate, 2 mM L-glutamine, pH7.4) for 1 h prior to the assay. OCR was measured with sequential injection of Oligomycin, FCCP and Rotenone/Antimycin (final concentration: 1, 1, and 0.5 µM, respectively). For ECAR detection, microplates were incubated with basic culture medium (containing 1 mM L-glutamine, without Glucose) for 1 h prior to the assay. ECAR was measured with sequential injection of glucose, oligomycin and 2-deoxyglucose (final concentration: 10 mM, 1 μM and 50 mM respectively).

### ^13^C-based metabolic flux analysis

ANKRD22-overexpressing RKO cells and* ANKRD22* knockdown HT-29 cells were cultured in glucose-free DMEM medium supplemented with 6 mM ^13^C_6_-glucose (Sigma), 10% fetal bovine serum, and 100 μg/ml of gentamicin for 4 h. At the end of incubation, media were removed; cells were washed with cold phosphate-buffered saline (PBS), and harvested using cell scrapers. The cells were resuspended in 1 mL of cold 80:20 methanol: H_2_O and vortexed for 1 min, repeatedly frozen and thawed 3 times in liquid nitrogen, and centrifuged at 12000 rpm/min for 10 min. The supernatant was dried under nitrogen, then resuspended in acetonitrile: H_2_O mixture (50:50) for LC-MS analysis. An ACQUITY UPLC BEH Amide column (1.7 μm, 2.1 mm×100 mm, Waters, USA) was used with mobile phase A (ultra-pure water containing 5 mM NH_4_Ac and 0.04% NH_4_OH), mobile phase B (95% acetonitrile +5% ultra-pure water containing 10 mM NH_4_Ac and 0.04% NH_4_OH). Gradient elution was with 10% mobile phase A + 90% mobile phase B for 0.1 min, 40% mobile phase A + 60% mobile phase B for 21 min. Elution rate was 0.3 mL/min, column temperature, 40°C, and the volume of sample was 2 μl. The analysis was performed as previously described 25.

### RNA extraction, RT-PCR, and RT-qPCR

Total RNA was extracted from cells by TRIZOL reagent (Macherey-Nagel, Germany). Extracted RNA was reverse-transcribed to cDNA using a PrimeScript™ RT reagent kit with gDNA Eraser (Takara, Japan) according to the manufacturer's instructions and then subjected to PCR amplification using Premix Ex Taq™ kit (Takara) (95℃ for 30 s, followed by 40 cycles of 95℃ for 5 s and 60℃ for 30 s) using a CFX Connect system (Bio-Rad, USA). The primers and probes were chemically synthesized by Sangon Biotech (China) and are listed in **Table S4**.

### Chromatin immunoprecipitation (ChIP)

The ChIP assay was performed by SimpleChIP Plus Enzymatic Chromatin IP Kit (Cell Signaling Technology) according to the manufacturer's instructions. In brief, SGC7901 cells were seeded in a 10 cm dish overnight and subsequently transiently transfected with the MAX/pCMV6-XL5 plasmid for an additional 48 h. The transfected cells were fixed with 1% formaldehyde, and the reaction was terminated by the glycine solution. Cells were lysed and chromatin was harvested and fragmented using enzymatic digestion. The ChIP assay was performed using anti-MAX antibodies and Protein G agarose beads. After protein-DNA de-crosslinking, DNA was purified using a DNA purification spin column. PCR was used for the detection of the ANKRD22 upstream DNA fragments with the primers: Forward: 5'-CCAGACACGTGTGGCTCTCA-3', Reverse: 5'-GGCAGGAAGGACTCACGGTT-3'. A diluted chromatin sample was used as an input. Chromatin fragments reacted with anti-Histone H3 antibody or normal rabbit IgG were used as a positive or negative control, respectively.

### Construction, production, and infection of recombinant lentivirus

The construction and production of target gene overexpression/knockdown recombinant lentivirus was entrusted to Cyagen Biosciences. For overexpression experiments, CRC cells were infected with recombinant lentivirus encoding ANKRD22, ANKRD22 fused with Halo-tag, or ANKRD22 fused with 3 tandem nuclear localization signals (5'-GATCCAAAAAAGAAGAG AAAGGTAGATCCAAAAAAGAAGAGAAAGGTAGATCCAAAAAAGAAGAGAAAGGTA GGATCCACCGGATCTAGA-3') for 48 h. Control cells were infected with corresponding blank vector lentivirus. For knockdown experiments, CRC cells were infected with lentivirus encoding shRNAs against *ANKRD22*、*SLC25A5*, or *ESYT1* for 48 h. Control cells were infected with scrambled lentivirus. Subsequently, cells were selected by 5 μg/ml puromycin for 14 days. The efficiency of overexpression or knockdown was examined by WB. When cells were infected with different lentiviruses for the second time, limiting dilution was applied to pick single clones, which were verified by WB. For transient overexpression and knockdown, cells were infected with the corresponding lentivirus for 72 h and used in subsequent experiments.

### Separation of nuclear and cytoplasmic fractions

Nuclear and cytoplasmic fractions were separated by chromatin extraction kit (Abcam). 1×10^6^ cells were harvested and washed with PBS twice, 200 μl of the lysis buffer was added; cells were kept on ice for 10 min, vortexed vigorously for 10 s, and centrifuged at 5000 rpm for 5 min. The supernatant was saved as the cytosolic fraction and 100 μl of extraction buffer was added to the precipitate, which was kept on ice for 10 min, vortexed occasionally, and sonicated 2×20 s. The samples were then centrifuged at 12,000 rpm, 4 ℃ for 10 min and 100 μl of chromatin buffer was added to the supernatant to obtain the nuclear fraction.

### Isolation of mitochondria and mitochondrial membrane

Mitochondria and cytoplasm were separated by mitochondrial separation kit (Beyotime, China) and mitochondrial matrix and membrane were separated by mitochondrial membrane separation kit (BestBio, China).

### Immunofluorescence co-localization analysis

CRC SW620, HT-29, and RKO cells were infected with lentivirus expressing Halo-ANKRD22 fusion protein, and pcDNA3.1(-) plasmid expressing Halo-ANKRD22 fusion protein was transfected into SGC-7901, NCI-H1299, and HeLa cells with Lipofectamine 3000 reagent (Invitrogen, USA). On the following day, the cells were transferred to a 24-well plate preloaded with cover glasses. On the third day, cells were washed with PBS and incubated with 5 μM HaloTag TMR Ligand at 37℃ for 30 min. Subsequently, the cells were washed with PBS 3 times and incubated in 150 nM MitoTracker™ Green FM (Invitrogen) for 30 min. Cells were then washed again with PBS 3 times and incubated in 5μg/ml Hoechst33342 dye for 15 min with final 3 washes with PBS. The cover glasses were taken out and observed under a confocal microscope (Carl Zeiss, Germany). To detect the sub-localization of ANKRD22 in organoid-cultured cells, SW620 and RKO cells overexpressing Halo-ANKRD22 fusion protein were cultured in Matrigel matrix for 7 days. After recovery, cells were placed onto slides, stained with Halo-tag TMR Ligand and Hoechst 33342 and treated as described above.

### Lipidomics

The analysis of lipidomics was performed as previously described 26, 27. Mitochondria were isolated from organoid-culture RKO cells overexpressing Halo-ANKRD22 or empty vector as a control. The relative abundance difference in lipids between the ANKRD22-overexpressing and control groups was presented by the ratio of lipid peak areas (ANKRD22/control). Lipids with a difference of over 100 times were listed on the heat map.

For the lipidomic analysis in mice tissues, mitochondria were isolated from mice colorectal tissues (*Ankrd22*^-/-^ vs wild-type C57BL/6) (n=6). Data were normalized and analyzed with multivariate statistical analysis using Simca-P 13.0 software. Principal component analysis (PCA) was first performed for unsupervised data analysis with UV data transformation. One abnormal sample W6 was deleted according to the PCA score map. Then, partial Least Squares-Discriminant Analysis (PLS-DA) and orthogonal-partial Least Squares-Discriminant Analysis (OPLS-DA) were performed using supervised data analysis. Differential lipids were identified by Student's *t*-test (*p*< 0.05) and fold-change values (|log 2 (fold change) | > 0.585).

### Determination of lactate production/glucose consumption ratio

1×10^6^ CRC cells and corresponding organoid-cultured cells were digested into single cells and cultured in fresh RPMI 1640 culture media for 4 h. The concentrations of lactic acid and glucose in the supernatant were measured. For evaluating the effect of ANKRD22 on lactate production/glucose consumption ratio of non-CCICs under TME, cells were cultured for 20 h under untreated condition, hypoxia (5% O_2_), low glucose (3mM glucose), or IFN-gamma (30ng/ml). Culture media were replaced by fresh media (RKO in MEM, HT29 in McCoy's 5A) and lactate and glucose in the media were measured at the end of 4 h by lactate dehydrogenase (LDH1) and hexokinase (HK) assays, respectively. Finally, lactate production/glucose consumption ratio was calculated.

### Measurement of ATP, ADP, and AMP

The levels of ATP, ADP, and AMP were determined by Promega detection kits (Catalog number: FF2000, V7001, V5011). BCA protein assay kit (Sangon Biotech, China) was used to determine protein concentration. ATP, ADP, and AMP values were normalized by protein concentration.

### Pyruvate dehydrogenase (PDH) activity assay

PDH activity was determined by the PDH Assay Kit (Sigma). Mitochondria of cells or tissues were extracted for the enzymatic reaction. A450 absorbance was measured with the microplate reader (SpectraMax i3x, Austria). The results were normalized by protein concentration.

### Halo-tag pull-down assay

The processes of the pulldown assay were carried out according to manufacturer's instructions of the Halo-tag complete pull-down system (Promega). Briefly, 1×10^7^ cells transiently or stably expressing Halo-ANKRD22 fusion protein were lysed with 300 μl RIPA lysis buffer (pH 7.5, 50 mM Tris-HCl, 150 mM NaCl, 1% Triton X-100, 0.1% sodium deoxycholate) containing protease inhibitor. The lysate was homogenized on ice for 30 times, centrifuged at 13,000 rpm for 5 min, and the supernatant was collected. The cell lysate was diluted to 1 ml with diluting buffer (pH 7.5, 100 mM Tris-HCl, 150 mM NaCl) and 10 μl of the diluted lysate was set aside as the pre-binding fraction (as input in Western blot). Then, the diluted lysate was reacted with HaloLink resin for 60 min at RT. Subsequently, the resin was washed 5 times with resin equilibration/wash buffer (pH 7.5, 100 mM Tris-HCl, 150 mM NaCl, 0.05% IGEPAL® CA-630). For elution, the resin was resuspended with 50 μl of 1% SDS elution buffer and incubated for 30 min with shaking at RT. Control cells were subjected to the same pull-down procedure, and the eluent was used as a negative control. Mass spectrometry identifications of eluted proteins were performed by a reverse-phase high-pressure liquid chromatography system coupled with an ion-trap mass spectrometer (LTQ, Thermo Fisher Scientific). Also, WB was performed to verify the results of mass spectrometry.

### Transmission electron microscopy

Cells or tissue samples were washed with PBS and fixed with 2.5% glutaraldehyde at 4℃ over-night following which samples were dehydrated, embedded, solidified, and sliced. Slides were stained with 3% uranyl acetate-lead citrate and observed under a transmission electron microscope (Hitachi H-7650). Six morphology-integral cells were randomly selected from each electron microscopy sample, and the number of mitochondria and the proportion of morphological changes in each cell were recorded.

### Detection of Ca^2+^ concentration

Cells or primary colorectal tissues from *Ankrd22*^-^/^-^ mice and wild-type C57BL/6 mice were digested into single cells and incubated in the buffer containing10 μmol/L fluo-4 (Invitrogen) and 0.02% Pluronic® F-127 (Sigma) at 37℃ for 1 h in the dark. Then the fluorescence intensity was detected by Fluorescence-activated cell sorting (FACS) Canto II flow cytometry.

### Tissue array

Tissue array specimens of CRC were obtained from the Cancer Institute of Zhejiang University and donated by Dr. Liu Xiyong (Hope Biotech, China). A total of 112 valid tissue samples were used for the analysis. Immunohistochemical staining was performed to detect the expression of ANKRD22, p53, and β-catenin in CRC tissue samples. The samples were classified as low or high expression groups according to the expression level of ANKRD22 and p53, and divided into 4 levels according to β-catenin expression.

### Gene set enrichment analysis (GSEA)

The datasets for GSEA are available in Gene Expression Omnibus (GEO accession GSE39582, https:// www.ebi.ac.uk/arrayexpress/experiments/E-GEOD-39582/).

### Statistical analysis

All results for continuous variables were presented as mean ± SD. Unpaired two-tailed student's t-test was used for comparison between two independent samples unless otherwise stated. The count samples were compared using the χ2 test. Data were analyzed using SPSS 23.0 statistical software. p<0.05 was considered statistically significant. Statistical plots were made by GraphPad Prism 7.0 software.

## Results

### A novel protein ANKRD22 involved in the reprogramming of CRC cells is regulated by TME

**Figure 1:**
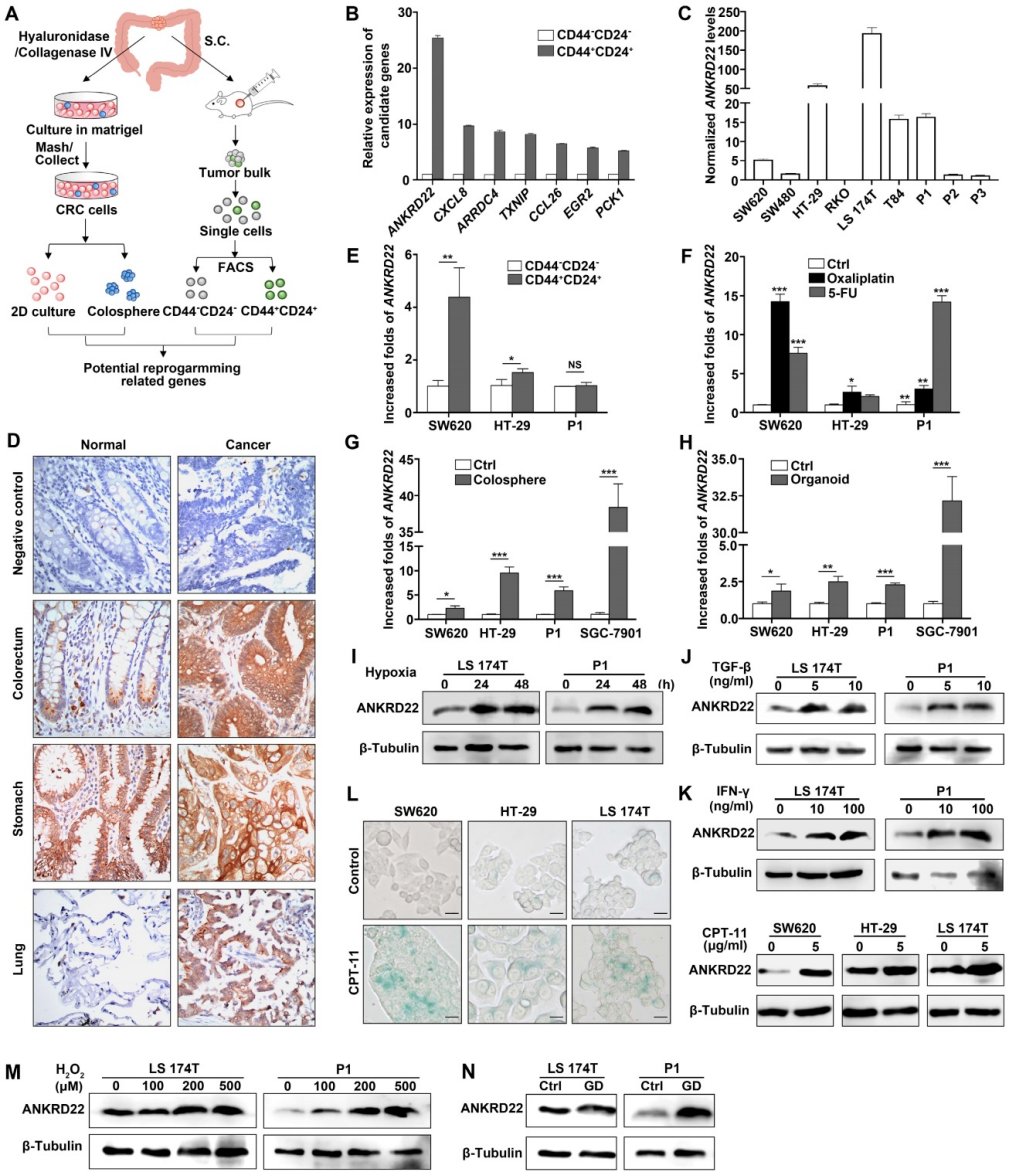
** The tumor microenvironment regulates a novel protein ANKRD22 involved in the reprogramming of CRC cells. (A)** Schematic strategy for screening reprogramming candidates regulated by the TME. S.C:Subcutaneous injection. **(B)** Quantitative determination of the candidate genes in CD44+/CD24+ and CD44-/CD24- subpopulations of human primary CRC cells by RT-qPCR. Data are presented as mean ± SD. **(C)** The level of *ANKRD22* in different CRC cell lines was determined by RT-qPCR. The levels of *ANKRD22* in CRC cell lines except RKO are presented as normalized relative folds to P3. **(D)** Immunohistochemistry staining of ANKRD22 in the human normal and cancerous epithelium of colorectum, stomach, and lung. Isotype IgG was used as the primary antibody in the negative control (Ctrl).** (E)** Determination of *ANKRD22* in CD44+/CD24+ and CD44-/CD24- CRC cells by RT-qPCR. **(F)** Determination of *ANKRD22* in drug-resistant CRC cells by RT-qPCR. CRC cells were cultured with or without (Ctrl) 2.5 μg/ml Oxaliplatin or 2.5 μg/ml 5-FU for 2 weeks. **(G)** Determination of *ANKRD22* in colosphere cells by RT-qPCR. Cells cultured in normal 2D condition were used as controls. **(H)** Determination of *ANKRD22* in organoid-cultured cells by RT-qPCR. Cells cultured in normal 2D condition were used as controls.** (I)** Detection of the effect of hypoxia on the expression of ANKRD22 in CRC cells. The hypoxia condition was built with GENbag (5% O_2_). **(J)** Detection of the effect of TGF-β on ANKRD22 expression. CRC cells were cultured in the medium supplemented with or without (Ctrl) 5 ng/ml TGF-β for 48 h. **(K)** Detection of the effect of IFN-γ on ANKRD22 expression. CRC cells were cultured in the medium supplemented with or without (Ctrl) 30 ng/ml IFN-γ for 48 h. **(L)** Detection of the effect of cell senescence on ANKRD22 expression. Cell senescence was induced by 5 μg/ml CPT-11 for 48 h. Senescence was identified by β-galactosidase staining. Scale bar: 20μm. **(M)** Detection of the effect of reactive oxygen species (ROS) on the expression of ANKRD22 in CRC cells. CRC cells were cultured in a medium supplemented with or without (Ctrl) different concentrations of H_2_O_2_ for 24 h. **(N)** Detection of the effect of glucose deprivation on the expression of ANKRD22 in CRC cells. CRC cells were cultured in a glucose-free medium (GD) or in a high-glucose (4.5 g/L glucose) DMEM medium (Ctrl) for 24 h. Data in 1E-H were presented as mean±SD and analyzed by Student's *t*-test. *p<0.05, **p<0.01, ***p<0.001, NS, not significant. The data of the control group were normalized to 1. *TUBB* was used as a relative quantitative internal reference.

TME promotes reprogramming of tumor cells and progression of CRC 5. To identify the molecules induced by TME that could promote CRC cell reprogramming, we first transplanted primary human CRC tissues subcutaneously into nonobese diabetic/SCID/IL-2Rgamma (null) (NSG) mice. The generated tumor mass was collected two months later and dispersed into individual tumor cells. FACS was used to sort the cells into CD24^+^CD44^+^ and CD24^-^CD44^-^ subpopulations. The differential expression of these two groups of cells was compared and analyzed by microarray-based global cDNA expression profiling. The same batch of primary CRC cells was cultured* in vitro* under a serum-free and low-adherence condition to enrich colosphere cells, and the differential expression was compared with those of the paired primary CRC cells. Candidate genes were screened according to the differential expression patterns identified by both *in vivo* and *in vitro* analyses (**Figure 1A**). Through this screening strategy, seven candidates were selected that might be regulated by the TME in primary CRC cells. We verified these genes by real-time quantitative PCR (RT-qPCR) and noted that *ANKRD22* showed the most prominent change among these candidates (**Figure 1B**). Given that the function of ANKRD22 was poorly understood, we chose to investigate this gene further. RT-qPCR and WB were performed to evaluate the expression of ANKRD22 in CRC cell lines. The results showed that, with the exception of RKO cells, ANKRD22 was expressed at different levels in various CRC cell lines (**Figure 1C, Figure S1A)**.

Next, we examined the distribution of ANKRD22 in different tissues by first evaluating its expression in normal and cancerous epithelial cells by immunohistochemical (IHC) staining. The results showed high expression of ANKRD22 in the normal human gastric epithelium, low expression of ANKRD22 in the normal human colorectal epithelium, and barely detectable levels of ANKRD22 in the normal human pulmonary epithelium (**Figure 1D**).

Although the expression of ANKRD22 was not significantly different between gastric cancer cells and the matched normal gastric epithelium, the expression of ANKRD22 was significantly elevated in CRC cells and pulmonary cancer cells compared with their normal epithelial counterparts (**Figure 1D**). These observations were consistent with the results of GEPIA (Gene Expression Profiling Interactive Analysis) and Oncomine data analysis (**Figure S1B-C**). Similarly, there was a progressively increasing trend in *ANKRD22* expression during the progression from normal colorectal epithelium to inflammatory bowel disease (IBD) to CRC **(Figure S1D).** We also analyzed the expression of ANKRD22 in CCICs enriched by FACS based on CD24+CD44+ markers, treatment with chemotherapeutic drugs, in serum-free medium with low-adherence, and organoid culture. The *ANKRD22* expression was significantly high in CCICs enriched by several methods mentioned above as determined by RT-qPCR (**Figure 1E-H**). These results indicated that the increased ANKRD22 in CCICs might be involved in the proliferation of initiating cells as well as carcinogenesis of the colorectal/pulmonary epithelium.

Finally, to determine whether the expression of ANKRD22 was affected by the TME, different TME conditions were simulated* in vitro* by subjecting the CRC cells to various stimuli. We found the expression of ANKRD22 was induced by various microenvironmental stimuli, including hypoxia, cytokines (TGF-β and IFN-γ), chemotherapeutic-agent-induced senescence, reactive oxygen species (ROS), and glucose deprivation (**Figure 1L-N**). However, stimulation with lactate did not affect the expression of ANKRD22 (**Figure S1E**). These results suggested that ANKRD22 is a novel protein regulated by TME and might be involved in the reprogramming of CRC cells.

### MAX as a sensor is involved in the transcription of ANKRD22 induced by TME

**Figure 2:**
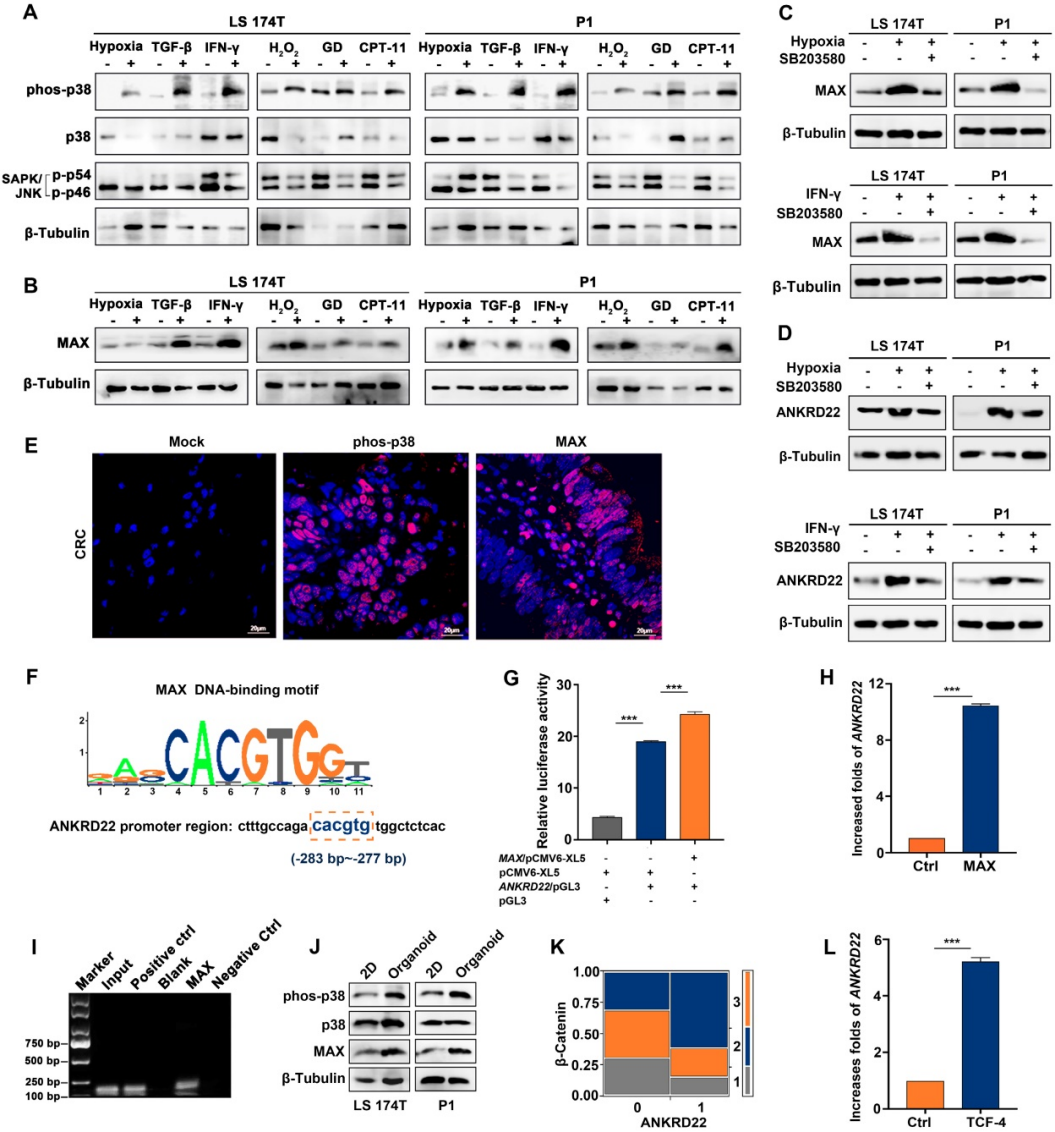
** MAX as a sensor is involved in the transcription of ANKRD22 induced by the tumor microenvironment. (A)** Detection of the activity of p38 and JNK pathways under TME stimulation by WB. CRC cells were cultured under stimulation of hypoxia (5% O_2_), 5 ng/ml TGF-β, 30 ng/ml IFN-γ, 5 μg/ml CPT-11 for 48 h or 500 μM H_2_O_2_, glucose deprivation for 24 h. **(B)** Detection of the expression of MAX in stimulated cells compared with normal cultured cells under TME stimulation by WB. **(C-D)**. Detection of the effect of p38 inhibition on the expression of MAX and ANKRD22 by WB. 10 μM p38 kinase inhibitor SB203580 was added to the cell culture system stimulated by hypoxia (5% O_2_) and 30 ng/ml IFN-γ for 48h. **(E)** Immunofluorescence detection of phosphorylated p38 and MAX expression in CRC tissues. Isotype IgG was used as the primary antibody in the negative control. **(F)** A potential binding site for MAX in the *ANKRD22* upstream promoter sequence. **(G)** The activity of the *ANKRD22* luciferase reporter after transfection of *MAX* into SGC-7901 cells. The results were analyzed by Student's *t*-test, ***p < 0.001. **(H)** Detection of the expression of *ANKRD22* after transfection of *MAX* into SGC-7901 by RT-qPCR. The results were analyzed by Student's* t*-test, **p<0.01. **(I)** ChIP analysis of the binding of MAX and upstream sequence of *ANKRD22*. ChIP was performed using the anti-MAX antibody and Protein G agarose beads. A diluted chromatin sample was used as an input. Chromatin fragments reacted with anti-Histone H3 antibody or normal rabbit IgG were used as a positive or negative control, respectively. **(J)** Comparison of expression of phos-38, p38, and MAX in organoid-cultured cells and normal 2D-cultured cells by WB. **(K)** Analysis of the correlation between ANKRD22 and β-Catenin in tissue-array. The data were analyzed by χ^2^ test. **p<0.01. **(L)** Detection of the effect of TCF-4 on *ANKRD22* expression in SGC-7901 cells with or without (Ctrl) TCF-4 overexpression by RT-qPCR. Data are displayed as mean±SD and analyzed by Student's *t*-test, ***p<0.001.

TME has complex components where different stimuli exist with different induction mechanisms. We observed that hypoxia-induced HIF-1α promoted the transcription of *ANKRD22* under anoxic environment (**Figure S2A**). It was of interest to investigate the possibility of the existence of a common mechanism for inducing ANKRD22 expression by different stimuli. To this end, we first analyzed the activity of the cellular stress pathways and found that p38 kinase showed significant activation under different simulated TME stimulations *in vitro* (**Figure 2A**). Furthermore, MAX, a downstream transcription factor of the p38 pathway, also showed significant upregulation (**Figure 2B**). When p38 kinase activity was blocked with specific inhibitors in the hypoxic and IFN-γ-stimulated models, MAX expression was significantly reduced, and ANKRD22 was also decreased (**Figure 2C-D**). Similarly, immunofluorescence staining revealed activation of p38 kinase and expression of MAX in CRC tissues (**Figure 2E**), suggesting that the p38/MAX pathway may be involved in the stress induced by different stimuli in the CRC microenvironment. We, therefore, examined whether MAX regulated the expression of ANKRD22. Bioinformatic analysis showed the presence of a potential MAX-binding sequence in the promoter region of *ANKRD22* (**Figure 2F**). Using a luciferase reporter containing the promoter region of *ANKRD22*, we found that co-transfected *MAX* could significantly increase the luciferase activity (**Figure 2G**). Similarly, transfection of *MAX* could also markedly increase the transcription of *ANKRD22* as shown by RT-qPCR (**Figure 2H**). Also, the ChIP assay confirmed the binding of MAX to the* ANKRD22* promoter region (**Figure 2I**).

We also detected increased p38 kinase activity in CCICs (**Figure 2J**), indicating that the p38 pathway may also be involved in the regulation of ANKRD22 in CCICs. Furthermore, ANKRD22 expression was correlated with β-Catenin in tissue-array IHC staining (**Figure 2K**), and transfection of *TCF4* promoted transcription of *ANKRD22* (**Figure 2L**). These results suggested that the p38/MAX pathway was involved in the regulation of *ANKRD22* induced by TME. MAX as a common "sensor" promoted the expression of ANKRD22 by different stimuli, and *ANKRD22* transcription was also induced by the p38/MAX and Wnt/β-Catenin pathways activated in CCICs.

### ANKRD22 is a novel mitochondrial membrane protein

**Figure 3:**
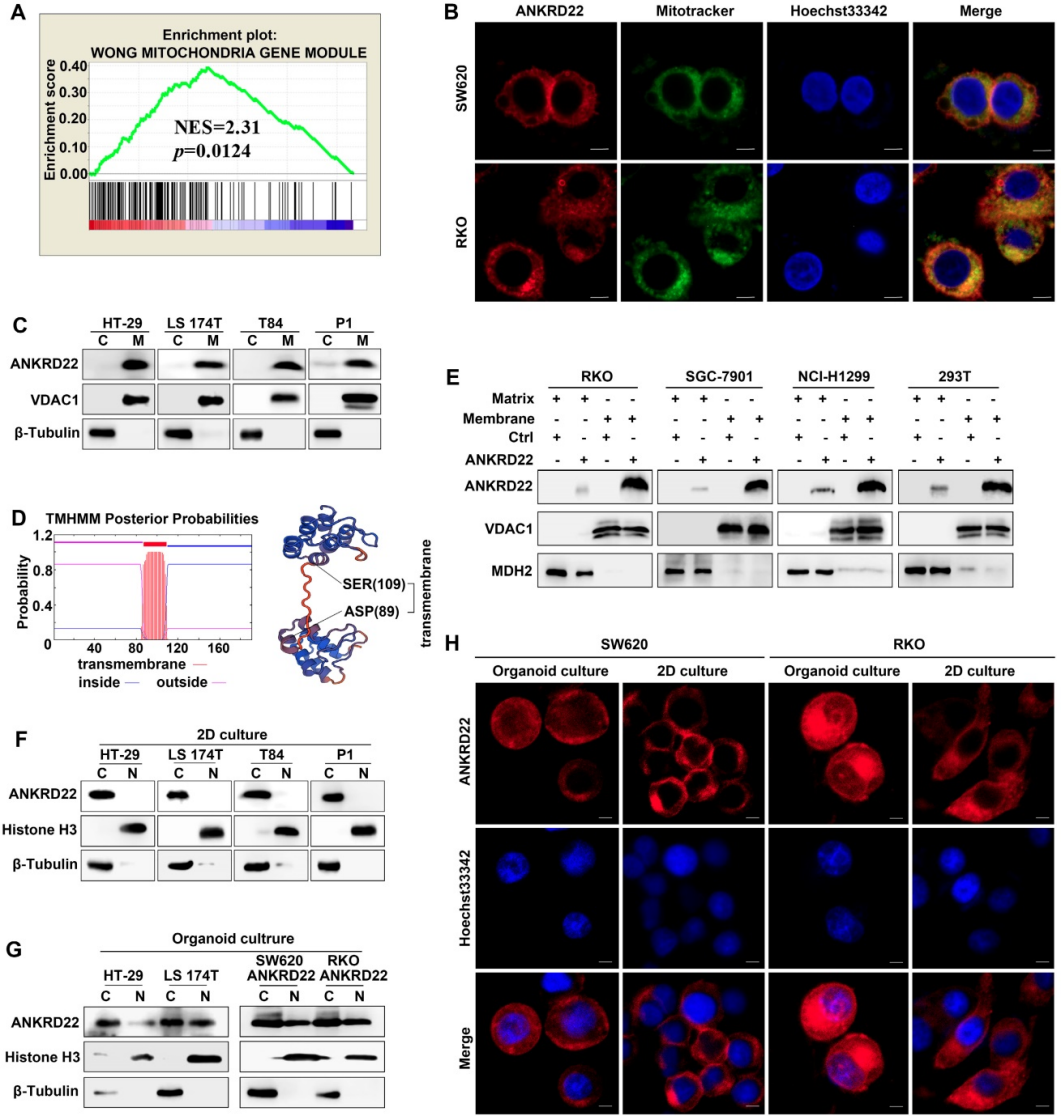
** ANKRD22 is a novel mitochondrial membrane protein. (A)** GSEA showed *ANKRD22* was enriched by a mitochondria-related gene set (Wong Mitochondria Gene Module), *p*< 0.05, probe: 238439_at. **(B)** Co-localization detection of exogenous-expressing ANKRD22 and mitochondria by confocal microscopy in colorectal cancer cells, Scale bar: 5μm.** (C)** Detection of ANKRD22 in mitochondria (M) and a residual cytoplasmic fraction (C) of cells ectopically expressing ANKRD22 by WB. β-Tubulin and VDAC1 were internal references for the cytoplasmic and mitochondrial fractions, respectively. **(D)** Transmembrane prediction (http://www.cbs.dtu.dk/services/TMHMM/) and spatial structure prediction (https://swissmodel. expasy.org/). **(E)** Detection of ANKRD22 distribution in the mitochondrial membrane and mitochondrial matrix of cells ectopically expressing ANKRD22 and in control cells by WB. VDAC1 and MDH2 were used as internal references for the mitochondrial membrane and mitochondrial matrix, respectively. **(F-G)** Detection of ANKRD22 distribution in CCICs. WB was used to detect the expression of ANKRD22 in the nucleus (N) and cytoplasm (C) of CRC cells in conventional 2D culture or organoid culture conditions. Histone H3 and β-Tubulin were used as nuclear and cytoplasmic internal references, respectively.** (H)** Detection of the subcellular localization of ANKRD22 in conventional 2D- or organoid-culture conditions, Scale bar: 5μm.

To determine the molecular characteristics of ANKRD22, we evaluated its organelle localization. First, we performed GSEA and observed that several mitochondrion-related genes were enriched in cells with a higher level of *ANKRD22* (**Figure 3A**), which suggested that the function of ANKRD22 might be related to mitochondria. Also, fluorescence colocalization analysis indicated that in colorectal, gastric, lung, and cervical cancer cells, almost all exogenous ANKRD22 was localized in mitochondria (**Figure 3B**, **Figure S3A**). WB analysis of mitochondria purified from CRC cells also showed that both exogenous and endogenous ANKRD22 was localized in mitochondria (**Figure 3C, Figure S3B**), suggesting that ANKRD22 is a novel mitochondrial protein. We predicted that ANKRD22 might be a mitochondrial membrane protein with a potential transmembrane region between amino acid positions at 87-109th (**Figure 3D**). We, therefore, isolated and purified the mitochondrial membrane and matrix and showed by WB that a major fraction of ANKRD22 was localized on the mitochondrial membrane, while a minor fraction was in the mitochondrial matrix (**Figure 3E**). Finally, we evaluated the potential change in the localization of ANKRD22 in CCICs. **(Figure 3F, Figure S3C)**, Fluorescence colocalization and WB analyses revealed that a small proportion of ANKRD22 was imported into the nucleus in CCICs different from its mitochondrial location in non-CCICs (**Figure 3G-H, Figure S3D**). Furthermore, GSEA showed that most enriched mitochondrial genes were related to metabolism and energy pathways, suggesting the involvement of ANKRD22 in the metabolism of CRC cells.

### ANKRD22 promotes aerobic glycolysis in CRC cells

**Figure 4:**
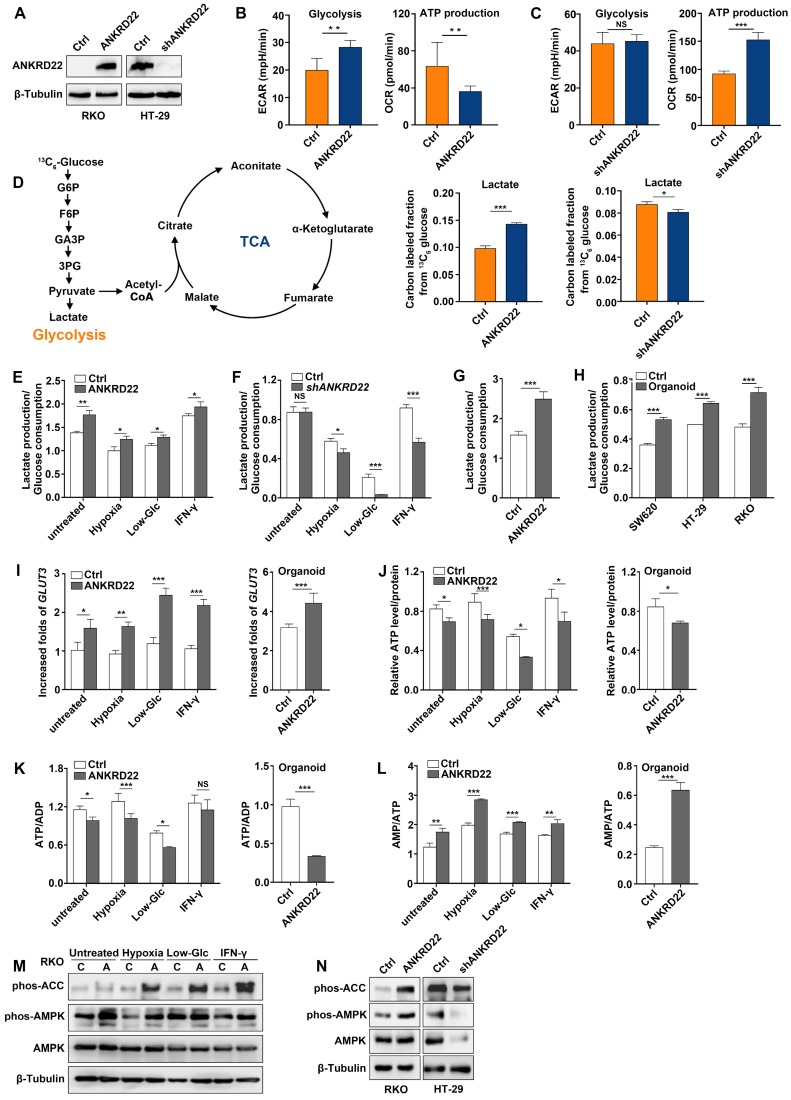
** ANKRD22 promotes aerobic glycolysis in colorectal cancer cells. (A)** Detection of the expression of ANKRD22 in ANKRD22-overexpressing cells or *ANKRD22*-knockdown cells by WB.** (B)** ECARs and OCRs were compared between ANKRD22-overexpressing RKO cells and control cells (Ctrl). Glycolysis levels and ATP production levels were analyzed by Student's *t-*test. **(C)** ECARs and OCRs were compared between* ANKRD22*-knockdown HT-29 cells and control HT-29 cells (Ctrl). **(D)** ANKRD22-overexpressing RKO cells and control cells, *ANKRD22*-knockdown HT-29 cells and control cells were labeled with ^13^C_6_-glucose. Diagram shows important intermediates in glycolysis and TCA cycle. The levels of metabolic intermediates were detected by LC-MS. The fraction of ^13^C isotope-labeled lactate are presented as mean±SD and analyzed by Student's *t-*test. **(E-F)** Effects of overexpression or knockdown of *ANKRD22* on the ratio of lactate production/glucose consumption under different TMEs (hypoxia, low-Glc, and IFN-γstimulation). **(G)** Effect of ANKRD22 overexpression on the ratio of lactate production/glucose consumption in organoid-cultured RKO cells.** (H)** Comparison of the ratio of lactate production/glucose consumption of organoid-cultured or conventional 2D condition-cultured CRC cells. **(I)** Determination of the effect of ANKRD22 overexpression on the expression of *GLUT3* under TMEs and in organoid-cultured RKO cells by RT-qPCR. Results were normalized to the value of control cells in the untreated group cultured under 2D condition. **(J)** Determination of the effect of ANKRD22 overexpression on cellular ATP levels under TMEs and in organoid-cultured RKO cells. **(K-L)** Determination of the effect of ANKRD22 overexpression on cellular ATP/ADP and AMP/ATP ratios under TMEs and in organoid-cultured RKO cells.** (M)** Determination of the effect of ANKRD22 overexpression on the activity of the AMPK pathway in RKO cells under TMEs. C: Control, A: ANKRD22 overexpression. **(N)** Determination of the effect of overexpression or knockdown of ANKRD22 on the activity of the AMPK pathway in organoid-cultured RKO and HT-29 cells. The data in this section are displayed as mean±SD and analyzed by Student's *t*-test, *p<0.05, **p<0.001, ***p<0.001, NS, not significant.

The above analyses suggested that ANKRD22 might participate in cellular energy metabolism. To define the metabolic alterations induced by ANKRD22, we first examined the effects of ANKRD22 overexpression and knockdown on OCR and ECAR. To compare its effects on glycolysis and OXPHOS, ANKRD22 was overexpressed in RKO cells that do not express it and was knocked down in HT-29 cells with its high expression **(Figure 4A)**. We found that overexpression of ANKRD22 significantly increased the ECAR associated with glycolysis and decreased the OCR associated with OXPHOS used for ATP production **(Figure 4B)**, while* ANKRD22* knockdown increased OCR, but its effect on ECAR was not obvious **(Figure 4C)**. Then, we further validated these findings by profiling cellular metabolism using ^13^C-based metabolic flux analysis. We found that ANKRD22 overexpression promoted the production of glycolytic intermediates, including ^13^C isotope-labeled glucose 6-phosphate (G6P), fructose 6-phosphate (F6P), glyceraldehyde 3-phosphate (GA3P), 3-phosphoglycerate (3PG) and lactate, while these intermediates decreased after knockdown **(Figure 4D, Figure S4A)**. In addition, ^13^C isotope-labeled pyruvate and tricarboxylic acid (TCA) cycle intermediates (citrate, aconitate, α-ketoglutarate, fumarate, malate) were largely decreased in ANKRD22-overexpressing cells** (Figure S4A-B)**. Results from the fraction of ^13^C-isotopologues suggested increased utilization of glucose through glycolysis and reduced utilization through TCA cycle.

To evaluate the effects of different microenvironmental factors on glycolysis, we used lactate production/glucose consumption ratio to measure glycolysis as previously reported 28. We selected hypoxia, low-glucose, and IFN-γ as TME stimulating factors and found that ANKRD22 overexpression promoted glycolysis both under the untreated condition and TME** (Figure 4E)**, while *ANKRD22* knockdown reduced the level of glycolysis only under microenvironmental stimulation** (Figure 4F)**. Similarly, overexpression of ANKRD22 in CCICs also promoted glycolysis **(Figure 4G).** Our results showed that CCICs have higher levels of glycolysis than non-CCICs** (Figure 4H)**, suggesting that ANKRD22 promotes metabolic reprogramming and initiating cell characteristics of CRC cells by promoting glycolysis. Increase in glycolytic phenotype requires an increase in glucose supply. Accordingly, we found that ANKRD22 overexpression induced transcription of *GLUT3* and *GLUT14* instead of* GLUT1* under TME stimulation **(Figure 4I, Figure S4C)**, while ANKRD22 promoted only* GLUT3* transcription in CCICs **(Figure 4I, Figure S4D)**. Conversely, under hypoxic condition, *ANKRD22* knockdown showed significant down-regulation of *GLUT3* and* GLUT14* transcription **(Figure S4E)**, indicating that GLUT3 is a key protein both in the TME and CCICs that promotes glucose transport by ANKRD22.

Finally, we examined the effect of ANKRD22 on the total energy status of CRC cells by analyzing the levels of ATP, ADP, and AMP in ANKRD22-overexpressing and -knockdown cells under different stimuli and in CCICs. The results showed that total ATP levels and ATP/ADP ratio were significantly decreased** (Figure 4J-K)**, whereas the AMP/ATP ratio and AMPK activity were increased in ANKRD22-overexpressing RKO cells after various stimulations and in CCICs **(Figure 4L-N)**. Knockdown of *ANKRD22* in CCICs also showed consistent inhibition of the AMPK activity** (Figure 4N)**. Together, these results indicated that ANKRD22 promotes aerobic glycolysis and possibly remodels the energy metabolism of CRC cells.

### ANKRD22 interacts with multiple targets in the glucose and ATP metabolism

**Figure 5:**
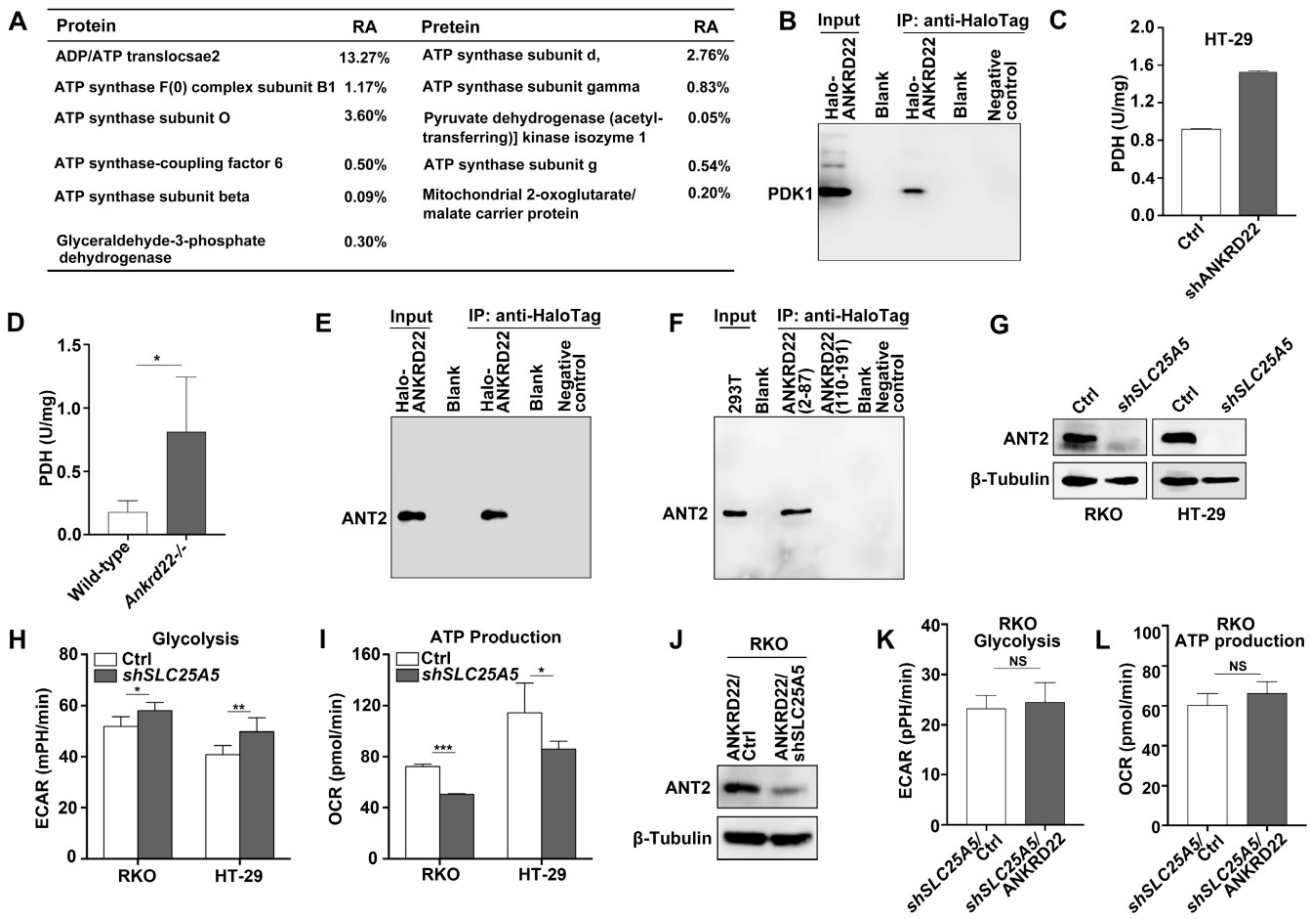
** ANKRD22 interacts with multiple targets in the glucose and ATP metabolism. (A)** The immunoprecipitation products of ANKRD22 related to energy metabolism detected by mass spectrometry. RA, Relative abundance. **(B)** Co-IP analysis of the interaction between ANKRD22 and PDK1. RKO cells stably expressing Halo-ANKRD22 fusion protein were analyzed with Co-IP according to the Halo-tag pull-down protocol. The fraction of the lysate before the binding was used as a positive control. Pull-down eluent of RKO cells infected with null lentivirus was used as a negative control. **(C)** Effect of *ANKRD22* knockdown on mitochondrial PDH activity. **(D)** Detection of PDH activity in the colorectal mitochondria of *Ankrd22*^-/-^ mice (n=3) and wild-type C57BL/6 mice (n=3). **(E)** Co-IP analysis of the interaction between ANKRD22 and ANT2. Halo-ANKRD22-expressing RKO cells were used for Co-IP assay according to the Halo-tag pull-down protocol. The fraction of the lysate before the binding was used as a positive control. Pull-down eluent of RKO cells infected with null lentivirus was used as a negative control. **(F)** Analysis of the interaction between two non-transmembrane fragments of ANKRD22 and ANT2. Fragment sequences fused with Halo-tag were transfected into 293T cells and Co-IP was performed. Lysate of 293T was used as a positive control. Pull-down eluent of 293T cells transfected with the empty vector was used as a negative control. **(G)** Detection of the expression of ANT2 in *SLC25A5*-knockdown RKO and HT-29 cells by WB. **(H)** Comparison of ECAR between* SLC25A5*-knockdown and control in RKO and HT-29 cells. **(I)** Comparison of OCR between* SLC25A5*-knockdown and control in RKO and HT-29 cells. **(J)** Detection of ANT2 expression by WB in ANKRD22-overexpressing RKO cells that were infected by* SLC25A5* shRNA or control lentivirus for 72h. **(K-L)** Comparison of ECAR and OCR between ANKRD22-overexpressing RKO cells and control RKO cells after knockdown of *SLC25A5*. Glycolysis and ATP production levels were analyzed by Student's *t*-test. Data are presented as mean±SD. *p<0.05, **p<0.01, ***p<0.001, NS, not significant.

To determine the molecular mechanism underlying the promotion of aerobic glycolysis by ANKRD22 in CRC cells, we utilized RKO cells with stable expression of Halo-ANKRD22 fusion protein. The results from pull-down assays coupled with mass spectrometry analysis found that more than 10 proteins, including the glycolysis “gatekeeper” pyruvate dehydrogenase kinase isoform 1 (PDK1), interacted with ANKRD22 (**Figure 5A, Table S1**). Subsequent co-immunoprecipitation (Co-IP) and WB experiments confirmed the binding of PDK1 to ANKRD22 (**Figure 5B**). Furthermore, we found that in both *ANKRD22* knockdown CRC cells and *Ankrd22^-/-^* mice, the enzymatic activity of PDH, the major glucose-metabolizing enzyme downstream of PDK1, was significantly upregulated after the silencing of *ANKRD22* (**Figure 5C-D, Figure S5A**). These results suggested that ANKRD22 might promote glycolysis by inhibiting the kinase activity of PDK1 in CRC cells. Unexpectedly, we also noted in the pull-down/mass spectrometry analyses that several ATP synthase subunits and ADP/ATP translocase 2 (ANT2, encoded by *SLC25A5)* that are downstream of glycolysis could also interact with ANKRD22, where ANT2 showed the highest abundance. The results from Co-IP and WB experiments verified the interaction between ANT2 and ANKRD22** (Figure 5E).** Following the construction of two predicted non-transmembrane fragments of ANKRD22, we confirmed that ANT2 was bound to the N-terminal fragment-containing amino acid residues 2-87 of ANKRD22 (**Figure 5F**). ECAR and OCR detection revealed that SLC25A5 silence slightly promoted glycolysis and inhibited OXPHOS in CRC cells (**Figure 5G-I**). It was remarkable that the effects of ANKRD22 on glycolysis and OXPHOS were abolished after silencing of *SLC25A5* in the ANKRD22-expressing cells (**Figure 5J-L**). These results suggested that ANKRD22 possibly remodels the energy metabolism of CRC cells at different levels by interacting with multiple targets in the mitochondria.

### Abnormal lipid accumulation in the mitochondria of CCICs with ANKRD22 expression

**Figure 6:**
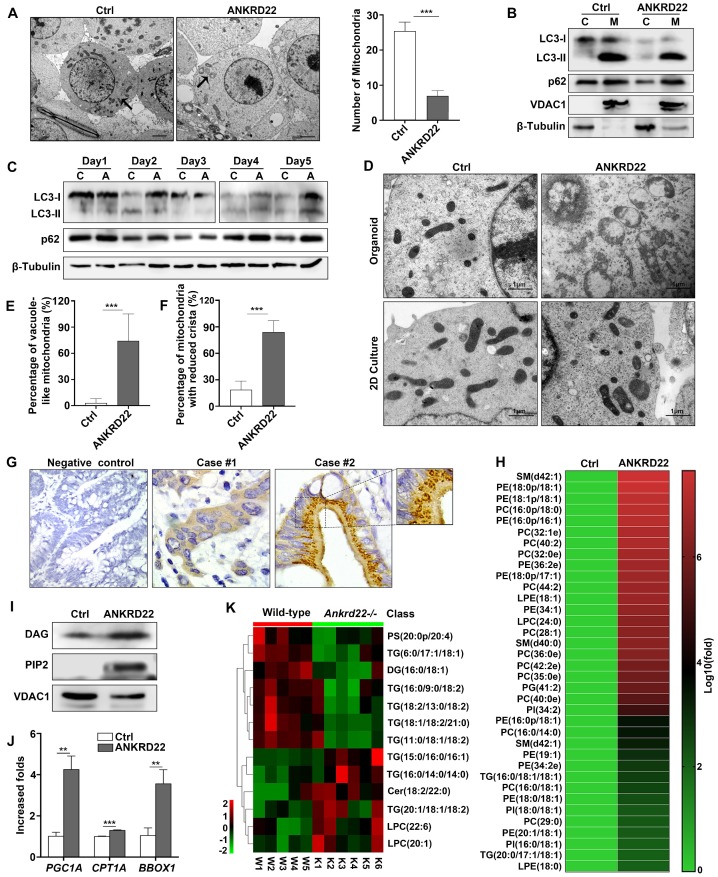
** Abnormal lipid accumulation in the mitochondria of CCICs expressing ANKRD22 (A)** The number of mitochondria decreased in ANKRD22-expressing cells. RKO cells stably expressing ANKRD22 and the empty vector were enriched by organoid culture and observed under a transmission electron microscope. Scale bar: 1 µm. The total number of mitochondria in each cell was counted (n=6). The data were analyzed by Student's *t*-test and are presented as mean±SD, ***p<0.001;** (B)** Detection of LC3 and p62 in the mitochondria of organoid-cultured RKO cells (ANKRD22 overexpression *vs.* control) by WB. β-Tubulin and VDAC1 were internal references for the cytoplasmic and mitochondrial fractions, respectively. **(C)** Effect of ANKRD22 on LC3 and p62 during organoid culture. RKO cells (ANKRD22 overexpression *vs.* control) were enriched by organoid culture for 1-5 days and then harvested and analyzed by WB.** (D-F)** Observation of mitochondria morphology. RKO cells (ANKRD22 overexpression *vs.* Control) were organoid cultured or cultured in conventional 2D conditions and observed under a transmission electron microscope. Scale bar: 1μm. The percentage of mitochondria with reduced vacuoles and crista in each cell was calculated (n=6). The data were analyzed by Student's *t*-test and are presented as mean±SD, ***p<0.001.** (G)** ANKRD22 staining in tissue array by IHC. Mitochondrial swelling existed in some of the patients (right), 40x.** (H)** Lipidomics assay of the mitochondria of organoid-cultured RKO cells. The relative abundance differences were calculated by the ratio of lipid peak area (ANKRD22 overexpression/control). Logarithmic processing was used to display the ratio in the heat map. **(I)** Detection of lipoprotein-binding PI (4,5) P_2_ and DAG in the mitochondria of organoid-cultured RKO cells (ANKRD22 overexpression *vs* control) by WB. **(J)** Determination of lipid oxidation-related enzymes (*PGC1A*, *CPT1A,* and *BBOX1*) in ANKRD22-overexpressing and control RKO cells by RT-qPCR. Cells were cultured as organoids. The data were analyzed by Student's *t*-test and are presented as mean±SD, **p<0.01, ***p<0.001. **(K)** Changes of lipid composition in the mitochondria of the colorectal epithelium in *Ankrd22^-/-^*mice (n=6) and wild-type C57BL/6 mice (n=5) as detected by lipidomics.

As a part of metabolic remodeling, previous studies showed that excess mitochondria must be removed during cell reprogramming 14, 21, 22. Herein, we demonstrated that ANKRD22 played a part in the promotion of glycolysis and reduction of ATP production in CRC cells. We then tested whether the expression of ANKRD22 affected the abundance and morphology of mitochondria. We found that the number of mitochondria in ANKRD22**-**expressing CCICs enriched by organoid culture was significantly lower compared with that in control cells as determined by electron microscopy (**Figure 6A**). However, neither electron microscopy (data not shown) nor WB analysis indicated that ANKRD22 could promote mitochondrial autophagy in the organoid culture-enriched CCICs or during their dynamic proliferation (**Figure 6B-C**). This finding suggested that mitochondrial autophagy might not be the primary mechanism for mitochondrial clearance by ANKRD22 during the reprogramming of CRC cells.

Surprisingly, electron microscopy revealed that the mitochondria in ANKRD22-expressing CCICs manifested prominent swelling and immature morphology with reduced cristae and a large number of vacuoles. The vacuoles appeared either on one side of the mitochondria or occupied the entire area (**Figure 6D-F**). Similarly, this mitochondrial degeneration was also found in tumor cells from patients (**Figure 6G**) but not in CRC cells following 2D culture (**Figure 6D**) or normal gastrointestinal epithelial cells (data not shown). To elucidate the cause of these mitochondrial changes in CCICs expressing ANKRD22, we analyzed the changes in mitochondrial content in the ANKRD22-expressing CCICs by lipidomics and WB. Analysis of Phosphatidylinositol 4,5-diphosphate2 (PIP2) and Diacylglycerol (DAG) of mitochondria showed that the lipid content was significantly higher in the mitochondria of ANKRD22-overexpressing CCICs than that in the control group (**Figure 6H-I**). In ANKRD22-overexpressing CCICs, the total ATP level and ATP/ADP ratio were markedly decreased while AMP/ATP ratio was increased **(Figure 4M-O)**, and the expression of fatty acid oxidation-related enzymes *PGC1*, *CPT1A* and *BBOX1* increased correspondingly** (Figure 6J)**, which could partially balance the loss in the number mitochondria.

Finally, to demonstrate whether ANKRD22 was involved in lipid metabolism in mitochondria *in vivo*, we examined the lipidomic changes in the colorectal epithelium of* Ankrd22*^-/-^ mice. Mitochondria were isolated from the colorectal epithelium to explore the effects of *Ankrd22*^-/-^ by mass spectrometry-based metabolomics. We detected significant changes in the mitochondrial lipid metabolites in the intestinal epithelia of mice (**Figure 6K**, **Fig S6A-B**) suggesting that ANKRD22 was involved in the lipid metabolism of mitochondria. Thus, the changes in abundance and morphology of mitochondria in CCICs may be related to the abnormal accumulation of lipids.

### Synergistic effect of ANKRD22 and E-Syt1 results in abnormal accumulation of lipids in the mitochondria of CCICs

**Figure 7:**
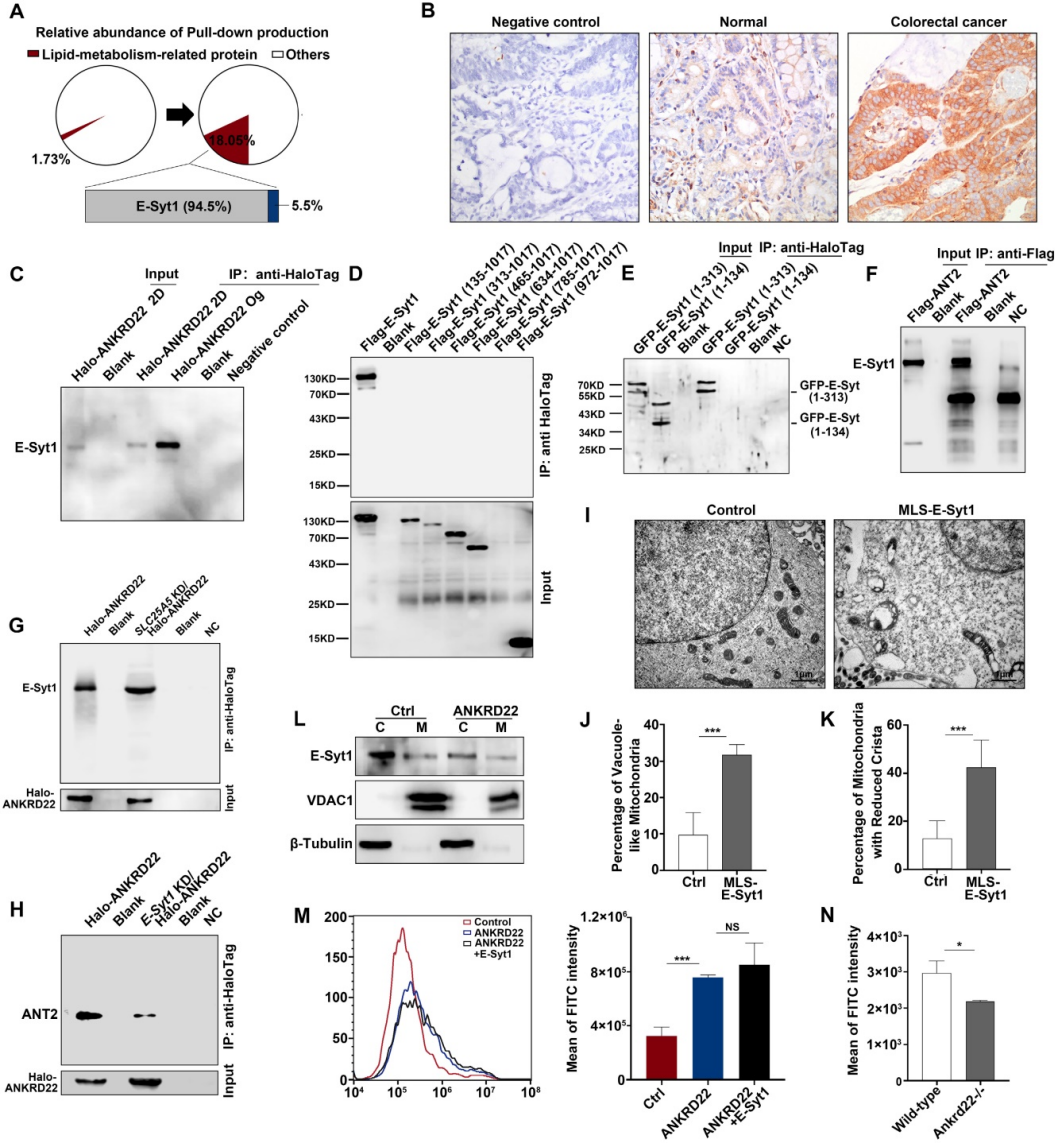
** Synergistic effect of ANKRD22 and E-Syt1 results in the abnormal accumulation of lipids in mitochondria of CCICs. (A)** Lipid metabolism-related proteins of the pull-down products of ANKRD22-overexpressing cells cultured in conventional 2D condition or enriched by organoid culture. **(B)** Expression of E-Syt1 in the normal colorectal epithelium and CRC epithelium by IHC. **(C)** Co-IP verification of the interaction between ANKRD22 and E-Syt1 in conventional 2D-cultured or organoid-cultured RKO cells with Halo-ANKRD22 overexpression. **(D-E)** Co-IP verification of the interaction between ANKRD22 and truncated E-Syt1s. Vectors encoding different regions of E-Syt1 were transfected into SGC-7901 cells that stably expressed Halo-ANKRD22 and performed Co-IP according to the Halo-tag pull-down protocol; the pull-down eluent of SGC-7901 cells was used as a negative control. **(F)** Co-IP verification of the interaction between ANT2 and E-Syt1. *Flag-SLC25A5*/pcDNA3.1(-) or pcDNA3.1(-) plasmids were transfected into E-Syt1-overexpressing 293T cells. Co-IP assay was conducted after 48 hours. **(G)** Influence of *SLC25A5* knockdown on the interaction between ANKRD22 and E-Syt1. *SLC25A5*-knockdown RKO cells and scrambled shRNA-infected RKO cells were infected with *Halo-ANKRD22* lentivirus, and the pull-down assay was performed; the pull-down eluent of scrambled shRNA-infected RKO cells was used as a negative control. **(H)** Influence of E-Syt1 knockdown on the interaction between ANKRD22 and ANT2. *ESYT1*-knockdown RKO cells and scrambled shRNA-infected RKO cells were infected with *Halo-ANKRD22* lentivirus and the pull-down assay was performed; the pull-down eluent of scrambled shRNA-infected RKO cells was used as a negative control. **(I-K)** Effect of mitochondria-localized E-Syt1 on mitochondrial morphology. An E-Syt1 sequence fused with a mitochondrial localization sequence at the C-terminus was transfected into ANKRD22-overexpressing 293T cells and observed by electron microscopy. The percentage of vacuole-like and crista-reduced mitochondria in each cell was calculated (n=6). **(L)** Detection of E-Syt1 in the mitochondria of organoid-cultured RKO cells (Halo-ANKRD22 overexpression vs control) by WB. β-Tubulin and VDAC1 were internal references for the cytoplasmic (C) and mitochondrial (M) fractions, respectively. **(M)**Effect of ANKRD22 and E-Syt1 on cytoplasmic Ca^2+^ level. Fluo-4 staining was used to detect the effect of ANKRD22 or synergistic effect of ANKRD22 and E-Syt1 on the cytoplasmic Ca^2+^ level of SGC-7901 cell *in vitro*. Three replicates were performed for each group. The fluorescence intensity of FITC was detected by FCM. The data were analyzed by Student's *t*-test and are presented as mean±SD, ***p<0.001. **(N)** Effect of *Ankrd22* knockout on cytoplasmic Ca^2+^ level. Primary colorectal cells of *Ankrd22^-/-^*mice (n=3) and wild-type C57BL/6 mice (n=3) were stained with fluo-4. The average fluorescence intensity was analyzed by Student's *t*-test, and data are presented as mean±SD, * *p*< 0.05.

To determine the ANKRD22-promoted lipid transport to the mitochondria and the molecular mechanism underlying the abnormal lipid accumulation, we first used mass spectrometry to compare the changes in the profiles of ANKRD22-interacting proteins of CCICs with those in their parental cells. The results revealed that the lipid transport protein, E-Syt1, was a highly enriched protein, potentially interacting with ANKRD22 in organoid cultures (**Figure 7A, Table S2**). E-Syt1 was ubiquitously expressed in the normal gastrointestinal epithelium and tumor cells with much higher expression in CRC cells compared with intestinal epithelial cells (**Figure 7B**, **Figure S7A**). The subsequent Co-IP experiments confirmed the interaction between ANKRD22 and E-Syt1 in CCICs. Although E-Syt1 could also be pulled down by ANKRD22 in non-CCICs, the amount was lower than in CCICs (**Figure 7C**). Transfection of truncated *E-Syt1* containing different domains followed by pull-down assays showed that ANKRD22 bound with E-Syt1 at the synaptotagmin-like mitochondrial lipid-binding protein (SMP)-LTD domain (**Figure 7D-E, Figure S7B**). We also identified amino acids 1-134 were essential for maintaining the binding conformation of ANKRD22 and E-Syt1. Co-IP experiments indicated that ANT2 also bound to E-Syt1, but the binding between ANKRD22 and E-Syt1 was independent of ANT2, whereas the presence of E-Syt1 enhanced the binding between ANT2 and ANKRD22 (**Figure 7F-H**).

Next, to verify the role of E-Syt1 in lipid accumulation and degeneration of mitochondria, we transfected *ESYT1* fused with a mitochondrial localization sequence (*MLS-ESYT1*) into ANKRD22-expressing 293T cells **(Figure S7C)**. Transmission electron microscopy revealed that, similar to CCICs overexpressing ANKRD22, the mitochondria of ANKRD22-expressing 293T cells manifested swelling, vacuolar-like changes, and reduced mitochondrial cristae after MLS-E-Syt1 overexpression (**Figure 7I-K).** However, E-Syt1-expressing 293T cells did not show similar changes **(Figure S7D)**. These findings suggested that E-Syt1 was a key target molecule for lipid accumulation and degeneration of mitochondria in an ANKRD22-dependent manner.

E-Syt1 is believed to be a protein anchored to the endoplasmic reticulum that can tether the endoplasmic reticulum to the plasma membrane for lipid exchange under the regulation of Ca^2+^. We, therefore, tested whether E-Syt1 could be localized to mitochondria and whether ANKRD22 affected intracellular Ca^2+^ concentration. To this end, we performed WB on the purified mitochondria and found that E-Syt1 was partly localized to mitochondria in CCICs (**Figure 7L**). We used flow cytometry (FCM) to examine the potential effects of ANKRD22, ANT2, and E-Syt1 on intracellular Ca^2+^ concentration. The results showed that the intracellular Ca^2+^ concentration was significantly increased after transfection with *ANKRD22*, whereas the expression of ANT2 and E-Syt1 had no significant effect on intracellular Ca^2+^ concentration (**Figure 7M**, **Figure S7E-F**). Consistent with these findings, Ca^2+^ concentration was significantly lower in the colorectal epithelial cells of *Ankrd22*^-/-^ mice (**Figure 7N**). These results suggested that the excess accumulation of lipids and degeneration of mitochondria in CCICs were due to the synergistic effects of ANKRD22 and E-Syt1.

### Nuclearly-translocated ANKRD22 interacts with p53

**Figure 8:**
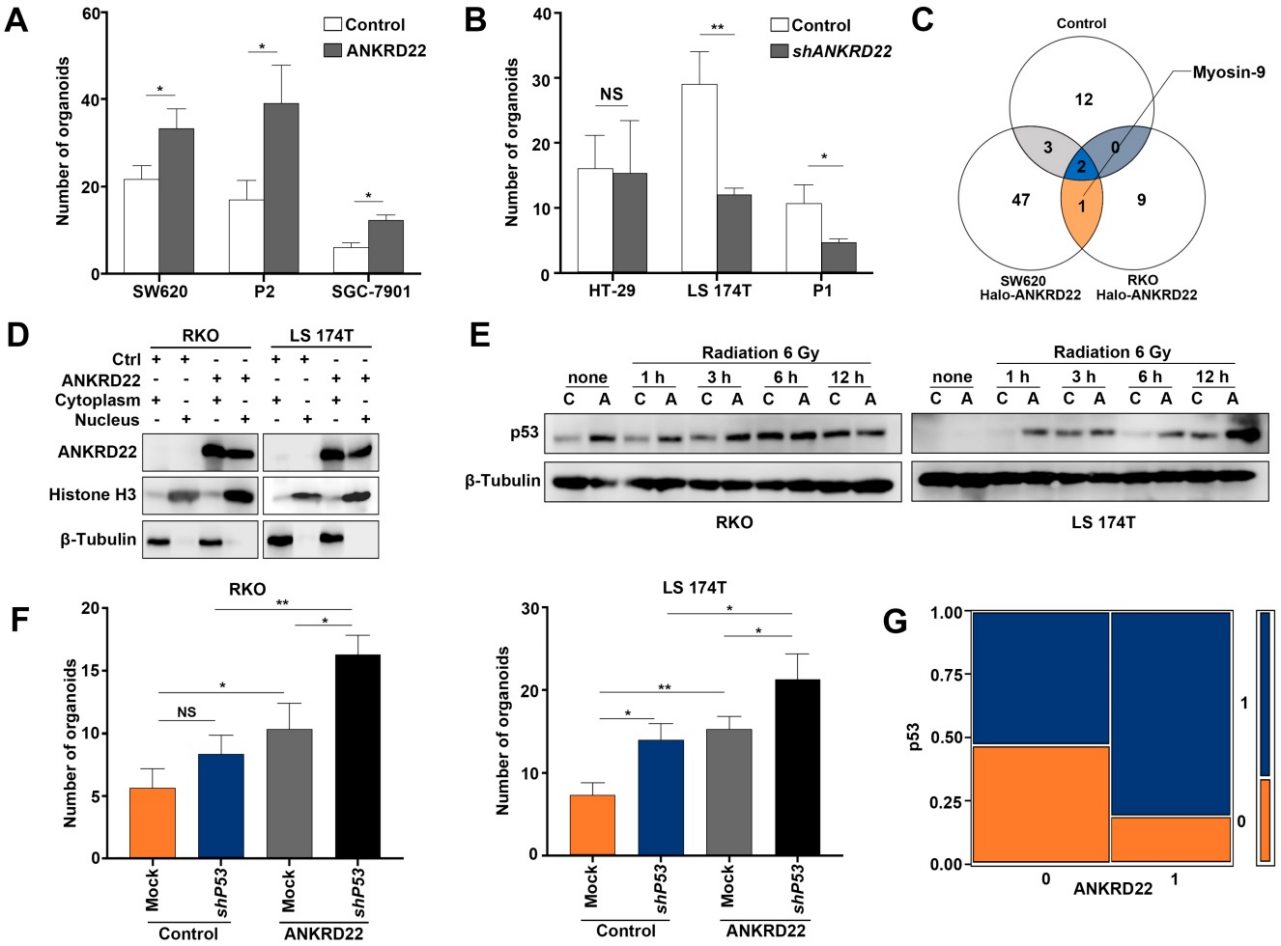
** Nuclearly translocated ANKRD22 interacts with p53. (A)** Quantification of organoid formation in ANKRD22-overexpressing cells. 100 ANKRD22-overexpressing cells (n=3) and control cells (n=3) were organoid cultured and counted one week later. The data were analyzed by Student's *t*-test and are presented as mean±SD, *p<0.05. **(B)** Quantification of organoid formation in *ANKRD22*-knockdown cells. 200 *ANKRD22* -knockdown (n=3) and control cells (n=3) were organoid cultured and counted one week later. The data were analyzed by Student's *t*-test and are presented as mean±SD, *p<0.05, NS, not significant. **(C)**Target-screening strategy of ANKRD22 in the nucleus. The schematic diagram shows the pull-down results of the nuclear fraction of SW620 and RKO cells that stably expressed Halo-ANKRD22, which were enriched by organoid culture. The pull-down eluent of the mixture of wild-type SW620 and RKO cells were used as a negative control.**(D)** Detection of ANKRD22 expression in the cytoplasm (C) and nucleus (N) of RKO and LS 174T cells, which were infected with lentivirus encoding ANKRD22 that fused 3 tandem nuclear localization sequences (3×NLS-ANKRD22) or empty vector lentivirus (Ctrl). Histone 3 and β-Tubulin were nuclear and cytoplasmic internal references, respectively. **(E)** Effect of ANKRD22 on wild-type p53. WB showed the expression of p53 in RKO and LS 174T cells with or without (Ctrl) ectopic expression of 3×NLS-ANKRD22. Cells were given 6 Gy radiation treatment and collected at different times post-irradiation. **(F)** Quantification of organoid formation in NLS-ANKRD22-overexpressing cells after *P53* knockdown. One hundred cells of each group were organoid cultured and counted one week later. Three replicates were performed for each group. The data were analyzed by Student's* t*-test and are presented as mean±SD, *p<0.05. **(G)** Analysis of the correlation between ANKRD22 and p53 in tissue-array. The data were analyzed by Student's *t*-test and are presented as mean±SD analyzed by χ^2^ test. **p<0.01.

It was of interest to examine whether ANKRD22 could promote the nuclear reprogramming of CRC cells. We, therefore, first evaluated the effect of ANKRD22 on the proliferation of CCICs and found that expression of ANKRD22 increased whereas the knockdown of *ANKRD22* reduced the number of CCICs (**Figure 8A-B, Figure S8A-B**). These observations suggested that ANKRD22 promoted nuclear reprogramming of CRC cells. Since ANKRD22 is also expressed in the normal gastrointestinal epithelium, especially in the stomach, it is intriguing that ANKRD22 does not promote the nuclear reprogramming of normal gastrointestinal epithelial cells. To this end, we further investigated the molecular target(s) of nuclear ANKRD22 involved in the reprogramming of CRC cells. Previous experiments showed that the primary partner proteins of ANKRD22 in the cytoplasm of CCICs were E-Syt1 and ANT2. However, E-Syt1 could not be imported into the nucleus (**Figure S8C)**, and nuclear ANT2 could not be co-immunoprecipitated by ANKRD22 (data not shown), despite its partial nuclear expression **(Figure S8D)**.

To search for other partner proteins of ANKRD22, we performed Co-IP and mass-spectrometric analyses of nuclear lysates of ANKRD22-expressing CCICs and found that Myosin-9 could be pulled down by ANKRD22 (**Figure 8C, Table S3**). Nuclear Myosin-9 has been shown to stabilize p53 and facilitate its nuclear accumulation 29, suggesting that ANKRD22 in the nucleus might affect the level of p53. We, therefore, investigated the effects of ANKRD22 on both wild-type and mutant p53. We established 3×Nuclear localization sequence (NLS)-ANKRD22-expressing CRC cells with wild-type p53 **(Figure 8D)**. We found 3×NLS-ANKRD22-expressing cells with higher levels of p53 after irradiation (**Figure 8E**). Furthermore, 3×NLS-ANKRD22-expressing CRC cells coupled with knockdown of wild-type *P53* significantly increased colony formation in the organoid-cultured CRC cells (**Figure 8F)**. On the other hand, tissue-array IHC staining of human CRC tissue also revealed that the expression of mutant p53 was higher in the group that highly expressed ANKRD22** (Figure 8G)**. These results suggested that ANKRD22 antagonized nuclear reprogramming of the normal gastrointestinal epithelium by stabilizing the amount of p53. However, this stabilization effect was not observed with mutant p53, and ANKRD22 even promoted nuclear reprogramming in synergy with mutant p53 in CRC cells.

## Discussion

In this study, we identified a novel protein, ANKRD22, which was induced by the TME through activation of p38/MAX pathway and associated with the reprogramming of colorectal cancer cells. ANKRD22 promoted glycolysis and reduced ATP levels by interacting with multiple targets in the glucose and ATP metabolism, accompanied by an increase of AMPK activity. ANKRD22 acted synergistically with E-Syt1 to promote excess lipid transport into the mitochondria. Our study suggests that the ANKRD22-mediated pathways represent a novel mechanism by which the TME promotes reprogramming of CRC cells.

ANKRD22 contains four ankyrin (ANK)-repeat motifs. Each ANK repeat motif has an L-shaped structure, with approximately 30-34 amino acid residues, consisting of two antiparallel α-helices and one β-hairpin and can form high-affinity molecular scaffolds 30, 31. Numerous mammalian proteins contain ANK motifs with diverse functions, including transcriptional regulation, cytoskeletal integrity, ion transport, signal transduction, inflammation, immunity, and tumorigenesis. As a nucleus-encoded mitochondrial protein, the function of ANKRD22 needs further investigation. Studies have shown that ANKRD22 is significantly increased in the macrophages of patients with an acute rejection reaction after a renal transplant 32 as well as in the peripheral blood mononuclear cells of patients with pancreatic cancer 33, in basal type I basal-like breast cancer 34, and in non-small cell lung cancer (NSCLC) 35. ANKRD22 can promote cell cycle progression by activating E2F1 and contributes to NSCLC progression 35. RNA sequencing has shown that *ANKRD22* is specifically expressed in cancer stem cells in Krebs-2 ascites 36, and analysis by RNAi library screening has revealed that ANKRD22 can reduce the reprogramming efficiency of normal mouse embryonic fibroblasts 37. However, the functions of ANKRD22 have not been studied in normal gastrointestinal epithelia, CRC, and CCICs.

In this study, we found that ANKRD22 functioned as a tumor suppressor in normal gastrointestinal epithelia by stabilizing the p53 protein. ANKRD22 was a frequent target that could be activated by various factors in the TME. The activation of the p38/MAX stress pathway was instrumental in the increased ANKRD22 induced by TME factors. This pathway was also partially involved in the regulation of ANKRD22 in addition to canonical Wnt pathway in CCICs. Also, the nuclear translocation of ANKRD22 in CCICs suggested that this protein could link metabolic reprogramming with nuclear reprogramming of CRC cells. Nevertheless, we did not find any NLS-related motif in the ANKRD22 sequence. MS analysis showed that ANKRD22 was not phosphorylated but was acetylated at lysine 166 and this modification was only detected in cytoplasmic ANKRD22. However, it appeared that ANKRD22 translocated into the nucleus in a lysine-166 acetylation independent manner (unpublished data).

The metabolic phenotype of CCICs is controversial, and different opinions exist about the dependence of CCICs on glycolysis or OXPHOS 38-40. We found CCICs possessed a higher level of glycolysis and ANKRD22-expressing cells showed metabolic changes similar to those in initiating cells. An increase in the level of glycolysis requires increased uptake of glucose. Among the various glucose transporters, GLUT1 and GLUT3 are thought to be most critical in increasing the metabolic rate 41. GLUT14 is a variant of GLUT3, and enhanced expression of GLUT14 has been reported in hypoxic CRC cells 42. In the process of promoting glycolysis by ANKRD22, we found that GLUT3 was the vital glucose transporter in TME and CCICs, and increased ANKRD22 promoted activation of the AMPK which was also activated by low-oxygen and glucose deprivation 43. These data suggested that although the ATP level was reduced during the conversion of metabolic phenotype to glycolysis by ANKRD22, the cells maintained the ATP balance by increasing glucose transport and activating AMPK. In different TMEs, we observed a positive correlation between ANKRD22 and glycolysis levels. Previous studies have suggested that different glucose concentrations affect the metabolic phenotype of cells 44. However, whether it was cultured in high glucose DMEM medium (25 mM glucose) or ATCC-recommended MEM (6 mM glucose), the effect of ANKRD22 on the level of glycolysis in RKO cells showed the same trend change in our experiments (data not shown). However, due to the complexity of microenvironmental factors in our current study, it was difficult to fully delineate the effects of ANKRD22 on glycolysis, energy metabolism, and AMPK activities in different TMEs, in particular, in *ANKRD22* knockdown cells. In the future, a comprehensive investigation is required to elucidate these mechanisms.

In exploring the mechanism by which ANKRD22 promoted glycolysis, we discovered many ANKRD22 target proteins involved in energy metabolism. Besides the gatekeeper of aerobic glycolysis, PDK1 and downstream PDH 45, ANT2 was identified as another target that bound to ANKRD22. ANT2 was shown to be mainly expressed in the inner membrane of mitochondria in undifferentiated and proliferating cells (including tumor cells) 37. It is generally believed that ANT2 is important for maintaining membrane potential in tumor cells, and inhibition of ANT2 activity can reduce cellular ATP levels and facilitate glycolysis 46, 47. We found that ANT2 was central to the role of ANKRD22 in glycolysis and OXPHOS. We also observed that ANKRD22 interacted with many subunits of ATP synthase. Mass spectrometry, as well as GSEA, showed enrichment of a large number of energy metabolism-related genes, including 11 ATP synthase subunits, in the cells highly expressing ANKRD22. Considering that ANT2, ATP synthase, and an inorganic phosphate carrier form the ATP synthase complex are involved in the transport of ATP/ADP and inorganic phosphate (Pi), we hypothesized that ANKRD22, PDK1, ANT2, and ATP synthase together form a macromolecular complex that co-regulates the energy metabolism of CRC cells. In this context, interactions of ANKRD22 with multiple targets restored the aerobic glycolysis level of CRC cells.

After the completion of metabolic reprogramming, the cells need to reduce the number of mitochondria and switch them to naive and immature morphology to adapt to the initiating cell state 48. We examined ANKRD22-expressing CCICs by electron microscopy and found a decreased number of mitochondria. Also, significant vacuole-like alterations occurred due to the accumulation of lipids in the mitochondria. These pathological changes were not observed in the crypt cells in normal gastrointestinal epithelia of humans and mice, suggesting that the lipid metabolism of CCICs was compromised. We also observed that ANKRD22 overexpression in CCICs promoted the expression of lipid oxidation-related enzymes (*PGC1A*, *CPT1A,* and *BBOX1*), which helped buffer the loss in mitochondria number and and lower ATP level.

We next discovered that mitochondrial translocation of E-Syt1 was a key molecular event of this pathological change. Transfection of *MLS*-*ESYT1* led to similar swelling and vacuolar changes in the mitochondria, pointing to a crucial role of E-Syt1 in this phenomenon. E-Syt1 contains one SMP region and five C2 regions and is anchored to the endoplasmic reticulum via the hairpin structure at the N-terminus 49. It forms a tether region that regulates the contact between the endoplasmic reticulum and plasma membrane 49, 50. Unlike E-Syt2/3, E-Syt1 can be induced by stress 50, 51. The binding of E-Syt1 to the plasma membrane is triggered by an increase in Ca^2+^ concentration.

In our study, we found that ANKRD22 and ANT2 promoted mitochondrial localization of E-Syt1, which enhanced the binding between ANKRD22 and ANT2, and ANKRD22 increased intracellular Ca^2+^ concentration to promote the lipid transport function of E-Syt1. Because ANKRD22 could bind to multiple subunits of ATP synthase, the changes in Ca^2+^ during this process could be the result of Na^+^-Ca^2+^ exchange. Hence, we propose that when Ca^2+^ is increased, E-Syt1 is activated and promotes the transport of lipids from the cytosol into the mitochondria, including DAG and PIP2. As a result, a large number of mitochondrial lipids are accumulated, accompanied by an increase in ability of lipid oxidation to compensate for the decrease in TCA cycle. Although studies suggested that E-Syt1 contributes to the formation of autophagosomes 52, we have not observed any autophagy caused by ANKRD22 in our experiments. This finding may imply that there could be an autophagy-independent mechanism responsible for mitochondrial clearance, and this mechanism may be closely related to lipid accumulation in mitochondria.

Finally, as is the case for other mitochondrial proteins, both ANKRD22 and ANT2 can be imported into the nucleus in CCICs. However, ANKRD22 in the nucleus does not bind to ANT2, nor does ANKRD22 participate in stem cell-related epigenomic changes, such as Prohibitin 53. We found that nuclear ANKRD22 interacted with p53. The stabilization of wild-type p53 by ANKRD22 may be mediated by Myosin-9, and the interaction between ANKRD22 and Myosin-9 may be related to the ATPase domain present in Myosin-9. These results further explain why ANKRD22 only induces glycolysis but not nuclear reprogramming in normal gastrointestinal epithelial cells.

Taken together, ANKRD22 is an important reprogramming-related protein in CRC. CCICs expressing ANKRD22 undergo lipid accumulation in the mitochondria and employ a novel mechanism of mitochondrial clearance, which depends on mitochondrial translocation of E-Syt1. The ANKRD22/E-Syt1 axis is a potential therapeutic target as it is central to the reprogramming of CRC cells induced by tumor environment.

## Supplementary Material

Supplementary figures and tables.

## Abbreviations

ANK: Ankyrin
ANT2: ADP/ATP translocase 2
CCIC: Colorectal cancer initiating cells
ChIP: Chromatin immunoprecipitation
CIC: Cancer initiating cells
Co-IP: Co-immunoprecipitation
CRC: Colorectal cancer
Ctrl: Control
DAG: Diacylglycerol
ECAR: Extracellular acidification rate
E-Syt1: Extended synaptotagmin-1
F6P: Fructose 6-phosphate
FACS: Fluorescence-activated cell sorting
FCM: Flow cytometry
G6P: glucose 6-phosphate
GA3P: Glyceraldehyde 3-phosphate
GD: Glucose deprivation
GSEA: Dene set enrichment analysis
IBD: Inflammatory bowel disease
IHC: Immunohistochemistry
MLS: Mitochondrial localization sequence
NLS: Nuclear localization sequence
NSCLC: Non-small cell lung cancer cells
NSG: Nonobese diabetic/SCID/IL-2Rgamma (null)
OCR: Oxygen consumption rate
OXPHOS: oxidative phosphorylation
3PG: 3-Phosphoglycerate
PDH: Pyruvate dehydrogenase
PDK1: Pyruvate dehydrogenase kinase isoform 1
PIP2: Phosphatidylinositol 4,5-diphosphate2
ROS: Reactive oxygen species
RT-qPCR: Real-time quantitative PCR
SMP: Synaptotagmin-like mitochondrial lipid-binding protein
TCA: Tricarboxylic acid
TME: Tumor environment
