# Supplementary material for: ANKRD22, a novel tumor microenvironment-induced mitochondrial protein promotes metabolic reprogramming of colorectal cancer cells
Source: Theranostics. 2020 Jan 1;10(2):516–36. doi: 10.7150/thno.37472 (PMC6929986; doi:10.7150/thno.37472)
Supplement: Supplementary file 1 — Supplementary figures and tables. [file thnov10p0516s1.pdf]

ANKRD22, a novel mitochondrial protein, induced by the tumor microenvironment promotes the metabolic reprogramming of colorectal cancer cells

Tianhui Pan[1,*], Jingwen Liu[1,*], Song Xu[1], Qiao Yu[1], Hongping Wang,[1] Hongxiang Sun[3] , Jia Wu[1], Yue Zhu[5] , Jianwei Zhou[2,#] and Yongliang Zhu[1,4,#]

Figure S1

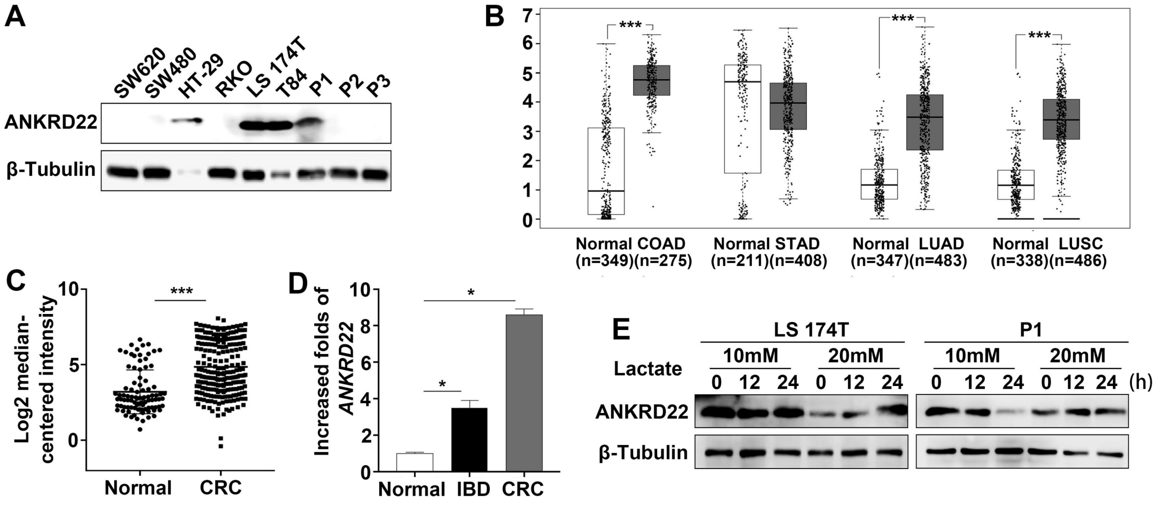

Figure S1 related to Figure 1. (A) Detection of the expression of ANKRD22 in different CRC cell lines by Western blot. **(B)** Expression of *A*NKRD22 in different tumors and corresponding normal tissues. Analysis was performed by GEPIA (http://gepia.cancer-pku.cn), of which RNA sequencing expression data were from the TCGA and the GTEx projects. COAD, Colon adenocarcinoma. STAD, Stomach adenocarcinoma. LUAD, Lung adenocarcinoma. LUSC, Lung squamous cell carcinoma. **(C)** Analysis of *ANKRD22* expression in CRC (n=241) and normal colorectal tissues (n=90) in 4 datasets from the Oncomine database. Data were analyzed by Student's *t*-test, ***p<0.001. **(D)** The relative expression of *ANKRD22* in normal colorectum (n=3), IBD (n=3) and CRC (n=3) epithelia measured by RT-qPCR. **(E)** Detection of the effect of lactate stimulation on the expression of ANKRD22 in CRC cell by Western blot.

Figure S2

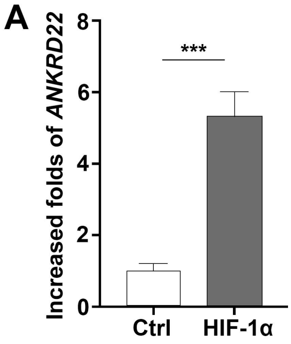

Figure S2 related to Figure 2. (A) Detection of the effect of HIF-1α overexpression on *ANKRD22* expression in SGC-7901 cells by RT-qPCR. Data were displayed as mean±SD and analyzed by Student's *t*-test, ****p*<0.001.

Figure S3

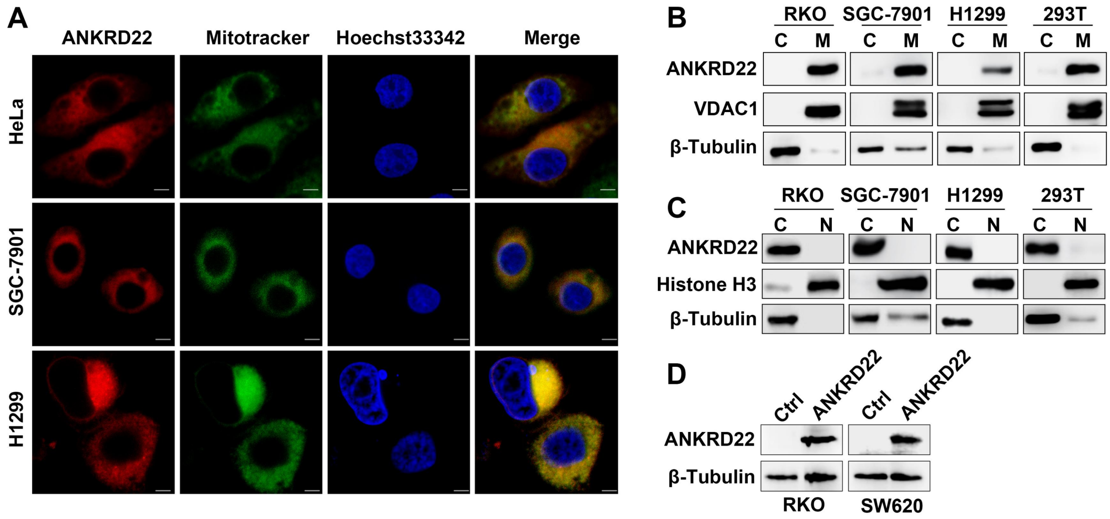

Figure S3 related to Figure 3. (A) Co-localization detection of exogenous-expressing ANKRD22 and mitochondria by confocal microscopy in HeLa, SGC-7901 and NCI-H1299 cells. Scale bar: 5μm. **(B)** Detection of exogenous-expressing ANKRD22 in the mitochondria (M) and residual cytoplasmic component **(C)** of cells by WB. β-Tubulin and VDAC1 were internal references for cytoplasmic and mitochondrial fractions, respectively. (C) Detection of exogenous-expressing ANKRD22 in the nucleus (N) and cytoplasm (C) of cells by WB. Histone 3 and β-Tubulin were internal references for nuclear and cytoplasmic fractions respectively. **(D)** Detection of ANKRD22 expression by WB in RKO and SW620 cells infected by lentivirus encoding Halo-ANKRD22 fusion protein. Cells infected by empty vector lentivirus were used as a control.

Figure S4

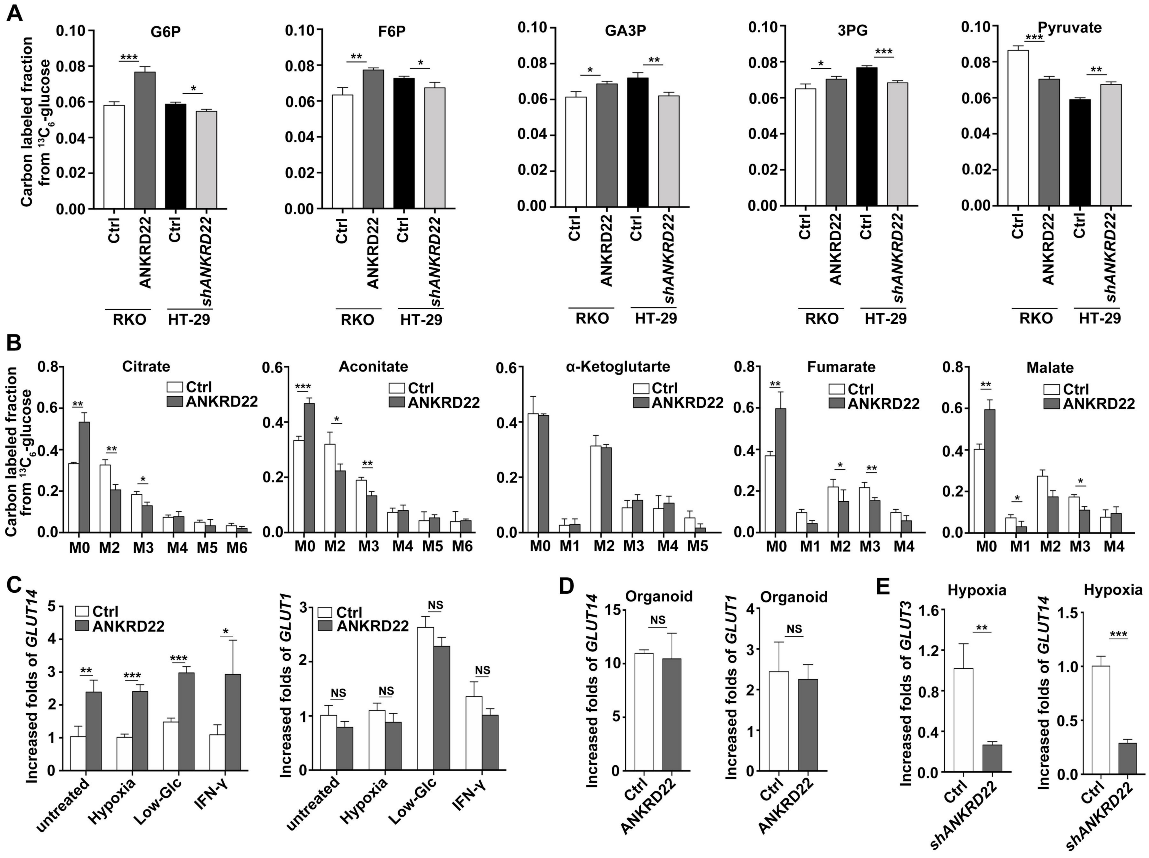

Figure S4 related to Figure 4. (A) The fraction of ^{13}C isotope-labeled metabolites in glycolysis, including G6P, F6P, GA3P, 3PG and pyruvate in ANKRD22-overexpressing RKO cells and control cells, *ANKRD22*-knockdown HT-29 cells and control cell. (B) The fraction of ^{13}C isotope-labeled metabolites in the TCA cycle, including citrate, aconitate, α-ketoglutarate, fumarate and malate in ANKRD22-overexpressing RKO cells and control cells. M0 to Mn isotopologues indicate the number of ^{13}C atoms present in each metabolite. (C) Determination of the effect of ANKRD22 overexpression on the expression of *GLUT14* and *GLUT1* under TMEs by RT-qPCR. (D) Determination of the effect of ANKRD22 overexpression on the expression of *GLUT14* and *GLUT1* in organoid-cultured RKO cells by RT-qPCR. Results in C and D were normalized to the values of control cells in the untreated group cultured under 2D condition. **(E)** Determination of the effect of ANKRD22 knockdown on the expression of *GLUT3* and *GLUT14* in HT-29 cells under hypoxia by RT-qPCR. Data in this figure were presented as mean±SD and analyzed by Student's *t*-test, *p<0.05, **p<0.01, ***p<0.001, NS, not significant.

Figure S5

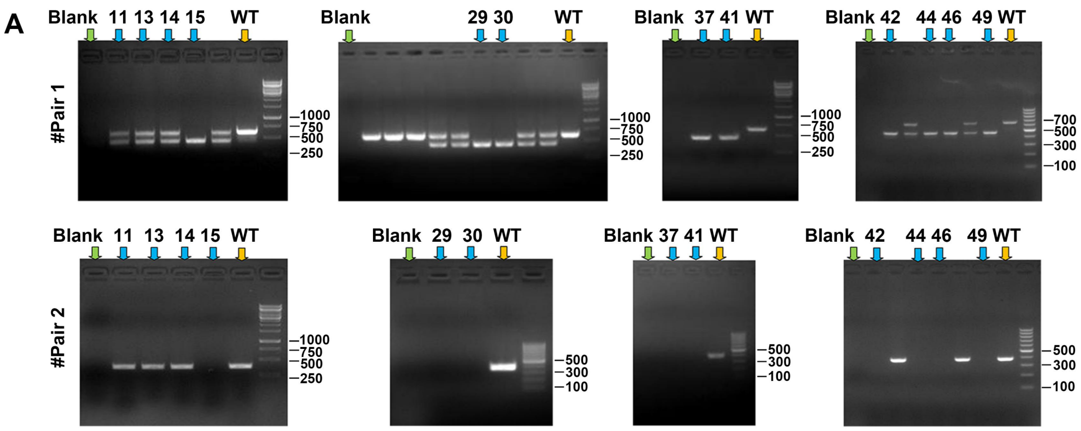

Figure S5 related to Figure 5. (A) The results of *Ankrd22* knockout of C57BL/6 mice were identified by PCR. Two different pairs of primers were used to amplify *Ankrd22*.

Figure S6

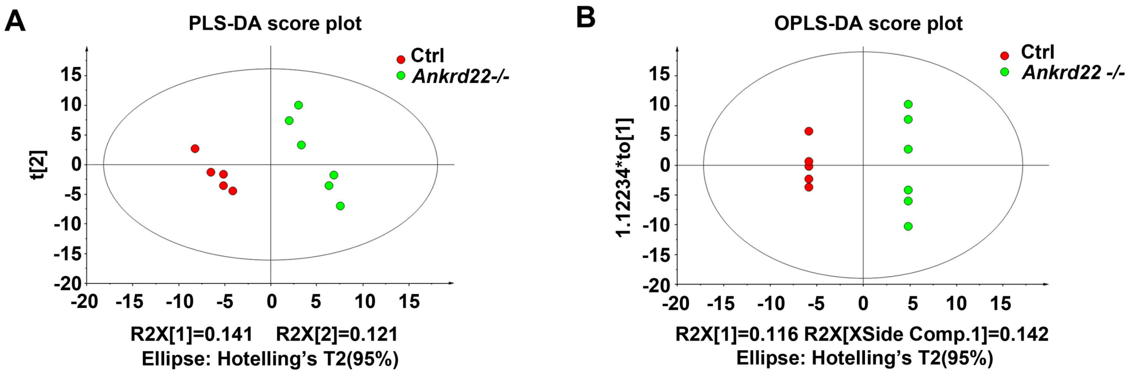

Figure S6 related to Figure 6. (A) PLS-DA score plot of *Ankrd22*$^{-/-}$(n=6) and Ctrl (n=5) group in lipidomics analysis. **(B)** OPLS-DA score plot of *Ankrd22*$^{-/-}$(n=6) and Ctrl (n=5) group in lipidomics analysis.

Figure S7

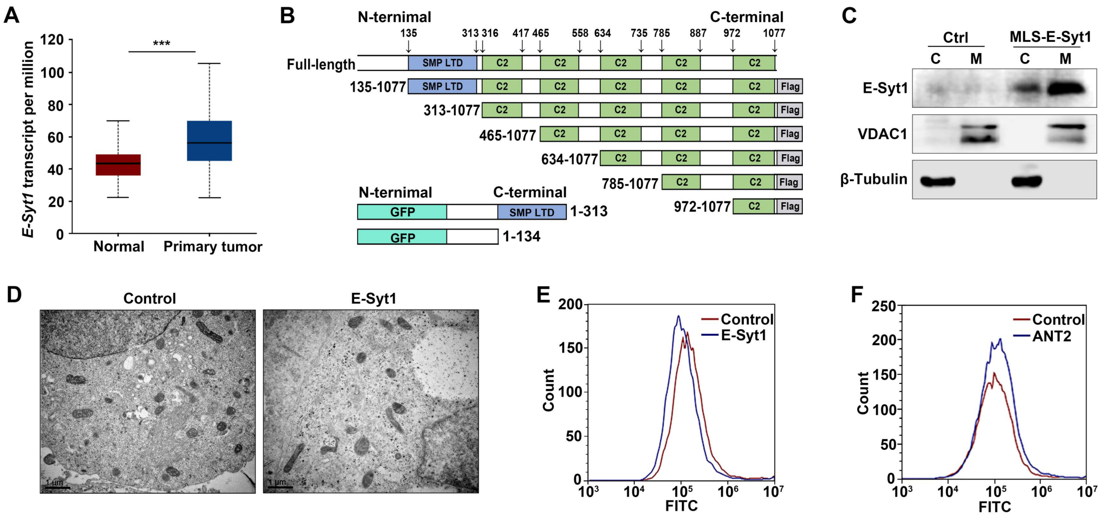

Figure S7 related to Figure 7. (A) *E-Syt1* expression in normal colorectal tissues (n=41) and CRC (n=286) analyzed by UALCAN website, ***p<0.001. **(B)** A schematic map of constructed eight different fragments of E-Syt1 based on its domains. 6 fragments fused with Flag tag at the C-terminal while 2 fragments fused with GFP tag at the N-terminal. **(C)** Detection of E-Syt1 expression in the mitochondria of 293T cells that were transfected with MLS-E-Syt1 or empty vector (Ctrl). β-Tubulin and VDAC1 were internal references for cytoplasmic (C) and mitochondrial (M) fractions, respectively **(D)** Observation of mitochondria morphology of 293T cells that were transfected with wild E-Syt1 or empty vector and observed under a transmission electron microscope. **(E-F)** Effect of E-Syt1 or ANT2 on cytoplasmic Ca^{2+} level. Fluo-4 staining was used to detect the effect of E-Syt1 or ANT2 on the cytoplasmic Ca^{2+} level of SGC-7901 cell. Fluorescence intensity of FITC was detected by FCM.

Figure S8

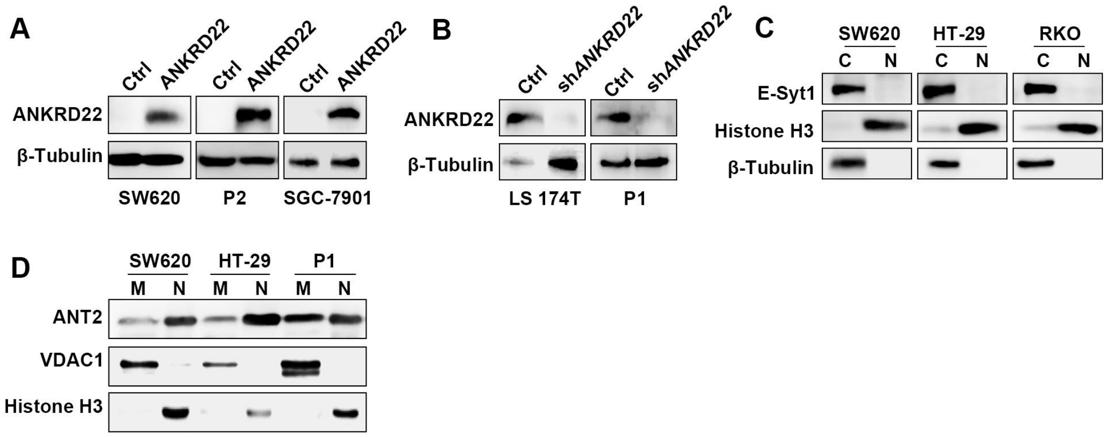

Figure S8 related to Figure 8. (A) Detection of ANKRD22 expression in gastrointestinal cells infected by lentivirus encoding ANKRD22 or empty vector lentivirus (Ctrl) by WB. **(B)** Detection of ANKRD22 expression in CRC cells infected by *ANKRD22* shRNA lentivirus or scrambled shRNA lentivirus (Ctrl) by WB. **(C)** Detection of E-Syt1 expression in the cytoplasm (C) and nucleus (N) of CRC cells by WB. Histone 3 and β-Tubulin were nuclear and cytoplasmic internal references respectively. **(D)** Detection of ANT2 expression in mitochondria (M) and nucleus (N) of CRC cells by WB. Histone 3 and VDAC1 were nuclear and mitochondrial internal references respectively.

Table S1

Pulldown proteins of ANKRD22 in 2D culture

Link	Pulldown molecules of ANKRD22		Pulldown molecules of negative control		Sequence Header
	Relative Abundance	Number of Peptides	Relative Abundance	Number of Peptides	
P04792	0.032093149	10	0	0	>sp|P04792|HSPB1_HUMAN Heat shock protein beta-1 OS=Homo sapiens GN=HSPB1 PE=1 SV=2
P05141	0.132670483	8	0	0	>sp|P05141|ADT2_HUMAN ADP/ATP translocase 2 OS=Homo sapiens GN=SLC25A5 PE=1 SV=7
Q71U36	0.043188367	8	0	0	>sp|Q71U36|TBA1A_HUMAN Tubulin alpha-1A chain OS=Homo sapiens GN=TUBA1A PE=1 SV=1
P18085	0.112377208	7	0	0	>sp|P18085|ARF4_HUMAN ADP-ribosylation factor 4 OS=Homo sapiens GN=ARF4 PE=1 SV=3
P23396	0.039478857	7	0	0	>sp|P23396|RS3_HUMAN 40S ribosomal protein S3 OS=Homo sapiens GN=RPS3 PE=1 SV=2
P07437	0.017000136	7	0	0	>sp|P07437|TBB5_HUMAN Tubulin beta chain OS=Homo sapiens GN=TUBB PE=1 SV=2
P62269	0.023538118	5	0	0	>sp|P62269|RS18_HUMAN 40S ribosomal protein S18 OS=Homo sapiens GN=RPS18 PE=1 SV=3
P62249	0.051934671	5	0	0	>sp|P62249|RS16_HUMAN 40S ribosomal protein S16 OS=Homo sapiens GN=RPS16 PE=1 SV=2
P60709	0.008775214	4	0	0	>sp|P60709|ACTB_HUMAN Actin, cytoplasmic 1 OS=Homo sapiens GN=ACTB PE=1 SV=1
O75947	0.027605297	4	0	0	>sp|O75947|ATP5H_HUMAN ATP synthase subunit d, mitochondrial OS=Homo sapiens GN=ATP5H PE=1 SV=3
P24539	0.011735538	4	0	0	>sp|P24539|AT5F1_HUMAN ATP synthase F(0) complex subunit B1, mitochondrial OS=Homo sapiens GN=ATP5F1 PE=1 SV=2
P51571	0.035619913	4	0	0	>sp|P51571|SSRD_HUMAN Translocon-associated protein subunit

					delta OS=Homo sapiens GN=SSR4 PE=1 SV=1
Q01650	0.003162564	3	0	0	>sp|Q01650|LAT1_HUMAN Large neutral amino acids transporter small subunit 1 OS=Homo sapiens GN=SLC7A5 PE=1 SV=2
P51149	0.014755368	3	0	0	>sp|P51149|RAB7A_HUMAN Ras-related protein Rab-7a OS=Homo sapiens GN=RAB7A PE=1 SV=1
Q00325	0.016866797	3	0	0	>sp|Q00325|MPCP_HUMAN Phosphate carrier protein, mitochondrial OS=Homo sapiens GN=SLC25A3 PE=1 SV=2
P36542	0.008280994	3	0	0	>sp|P36542|ATPG_HUMAN ATP synthase subunit gamma, mitochondrial OS=Homo sapiens GN=ATP5C1 PE=1 SV=1
Q5VYY1	0.009275611	3	0	0	>sp|Q5VYY1|ANR22_HUMAN Ankyrin repeat domain-containing protein 22 OS=Homo sapiens GN=ANKRD22 PE=2 SV=1
P62263	0.020528619	3	0	0	>sp|P62263|RS14_HUMAN 40S ribosomal protein S14 OS=Homo sapiens GN=RPS14 PE=1 SV=3
Q8WY22	0.031777639	3	0	0	>sp|Q8WY22|BRI3B_HUMAN BRI3-binding protein OS=Homo sapiens GN=BRI3BP PE=1 SV=1
P51148	0.018565728	3	0	0	>sp|P51148|RAB5C_HUMAN Ras-related protein Rab-5C OS=Homo sapiens GN=RAB5C PE=1 SV=2
P48047	0.036047137	3	0	0	>sp|P48047|ATPO_HUMAN ATP synthase subunit O, mitochondrial OS=Homo sapiens GN=ATP5O PE=1 SV=1
P62913	0.010767888	2	0	0	>sp|P62913|RL11_HUMAN 60S ribosomal protein L11 OS=Homo sapiens GN=RPL11 PE=1 SV=2
O95816	0.00299205	2	0	0	>sp|O95816|BAG2_HUMAN BAG family molecular chaperone regulator 2 OS=Homo sapiens GN=BAG2 PE=1 SV=1
P42677	0.040577173	2	0	0	>sp|P42677|RS27_HUMAN 40S ribosomal protein S27 OS=Homo sapiens GN=RPS27 PE=1 SV=3

P51151	0.00527008	2	0	0	>sp|P51151|RAB9A_HUMAN Ras-related protein Rab-9A OS=Homo sapiens GN=RAB9A PE=1 SV=1
P61026	0.009804593	2	0	0	>sp|P61026|RAB10_HUMAN Ras-related protein Rab-10 OS=Homo sapiens GN=RAB10 PE=1 SV=1
Q9NR31	0.055170446	2	0	0	>sp|Q9NR31|SAR1A_HUMAN GTP-binding protein SAR1a OS=Homo sapiens GN=SAR1A PE=1 SV=1
O60762	0.009630416	2	0	0	>sp|O60762|DPM1_HUMAN Dolichol-phosphate mannosyltransferase subunit 1 OS=Homo sapiens GN=DPM1 PE=1 SV=1
Q969X5	0.004517752	2	0	0	>sp|Q969X5|ERGI1_HUMAN Endoplasmic reticulum-Golgi intermediate compartment protein 1 OS=Homo sapiens GN=ERGIC1 PE=1 SV=1
Q15118	0.000472479	2	0	0	>sp|Q15118|PDK1_HUMAN [Pyruvate dehydrogenase (acetyl-transferring)] kinase isozyme 1, mitochondrial OS=Homo sapiens GN=PDK1 PE=1 SV=1
Q07021	0.012608879	2	0	0	>sp|Q07021|C1QBP_HUMAN Complement component 1 Q subcomponent-binding protein, mitochondrial OS=Homo sapiens GN=C1QBP PE=1 SV=1
P61224	0.001925609	1	0	0	>sp|P61224|RAP1B_HUMAN Ras-related protein Rap-1b OS=Homo sapiens GN=RAP1B PE=1 SV=1
P11441	0.002690838	1	0	0	>sp|P11441|UBL4A_HUMAN Ubiquitin-like protein 4A OS=Homo sapiens GN=UBL4A PE=1 SV=1
P67812	0.001898596	1	0	0	>sp|P67812|SC11A_HUMAN Signal peptidase complex catalytic subunit SEC11A OS=Homo sapiens GN=SEC11A PE=1 SV=1
P62829	0.003130231	1	0	0	>sp|P62829|RL23_HUMAN 60S ribosomal protein L23 OS=Homo sapiens GN=RPL23 PE=1 SV=1

<table>
<tr><td>P62987</td><td>0.008166933</td><td>1</td><td>0</td><td>0</td><td>>sp|P62987|RL40_HUMAN Ubiquitin-60S ribosomal protein L40 OS=Homo sapiens GN=UBA52 PE=1 SV=2</td></tr>
<tr><td>P60866</td><td>0.004948391</td><td>1</td><td>0</td><td>0</td><td>>sp|P60866|RS20_HUMAN 40S ribosomal protein S20 OS=Homo sapiens GN=RPS20 PE=1 SV=1</td></tr>
<tr><td>Q9GZT3</td><td>0.00483787</td><td>1</td><td>0</td><td>0</td><td>>sp|Q9GZT3|SLIRP_HUMAN SRA stem-loop-interacting RNA-binding protein, mitochondrial OS=Homo sapiens GN=SLIRP PE=1 SV=1</td></tr>
<tr><td>P18859</td><td>0.005039536</td><td>1</td><td>0</td><td>0</td><td>>sp|P18859|ATP5J_HUMAN ATP synthase-coupling factor 6, mitochondrial OS=Homo sapiens GN=ATP5J PE=1 SV=1</td></tr>
<tr><td>O75964</td><td>0.005431206</td><td>1</td><td>0</td><td>0</td><td>>sp|O75964|ATP5L_HUMAN ATP synthase subunit g, mitochondrial OS=Homo sapiens GN=ATP5L PE=1 SV=3</td></tr>
<tr><td>P62805</td><td>0.004814516</td><td>1</td><td>0</td><td>0</td><td>>sp|P62805|H4_HUMAN Histone H4 OS=Homo sapiens GN=HIST1H4A PE=1 SV=2</td></tr>
<tr><td>P02649</td><td>0.010311003</td><td>1</td><td>0.729752771</td><td>2</td><td>>sp|P02649|APOE_HUMAN Apolipoprotein E OS=Homo sapiens GN=APOE PE=1 SV=1</td></tr>
<tr><td>P0DMV9</td><td>0.001435031</td><td>1</td><td>0</td><td>0</td><td>>sp|P0DMV9|HS71B_HUMAN Heat shock 70 kDa protein 1B OS=Homo sapiens GN=HSPA1B PE=1 SV=1</td></tr>
<tr><td>P06576</td><td>0.00091116</td><td>1</td><td>0</td><td>0</td><td>>sp|P06576|ATPB_HUMAN ATP synthase subunit beta, mitochondrial OS=Homo sapiens GN=ATP5B PE=1 SV=3</td></tr>
<tr><td>Q5VTE0</td><td>0.001489542</td><td>1</td><td>0</td><td>0</td><td>>sp|Q5VTE0|EF1A3_HUMAN Putative elongation factor 1-alpha-like 3 OS=Homo sapiens GN=EEF1A1P5 PE=5 SV=1</td></tr>
<tr><td>P31689</td><td>0.000862834</td><td>1</td><td>0</td><td>0</td><td>>sp|P31689|DNJA1_HUMAN DnaJ homolog subfamily A member 1 OS=Homo sapiens GN=DNAJA1 PE=1 SV=2</td></tr>
<tr><td>Q96G23</td><td>0.00068181</td><td>1</td><td>0</td><td>0</td><td>>sp|Q96G23|CERS2_HUMAN Ceramide synthase 2 OS=Homo sapiens GN=CERS2 PE=1 SV=1</td></tr>
</table>

<table>
<tr><td>O14828</td><td>0.001694094</td><td>1</td><td>0</td><td>0</td><td>>sp|O14828|SCAM3_HUMAN Secretory carrier-associated membrane protein 3 OS=Homo sapiens GN=SCAMP3 PE=1 SV=3</td></tr>
<tr><td>P59190</td><td>0.008557488</td><td>1</td><td>0</td><td>0</td><td>>sp|P59190|RAB15_HUMAN Ras-related protein Rab-15 OS=Homo sapiens GN=RAB15 PE=1 SV=1</td></tr>
<tr><td>Q6IAN0</td><td>0.001042671</td><td>1</td><td>0</td><td>0</td><td>>sp|Q6IAN0|DRS7B_HUMAN Dehydrogenase/reductase SDR family member 7B OS=Homo sapiens GN=DHRS7B PE=1 SV=2</td></tr>
<tr><td>Q9UNL2</td><td>0.050330095</td><td>1</td><td>0</td><td>0</td><td>>sp|Q9UNL2|SSRG_HUMAN Translocon-associated protein subunit gamma OS=Homo sapiens GN=SSR3 PE=1 SV=1</td></tr>
<tr><td>Q02978</td><td>0.001982476</td><td>1</td><td>0</td><td>0</td><td>>sp|Q02978|M2OM_HUMAN Mitochondrial 2-oxoglutarate/malate carrier protein OS=Homo sapiens GN=SLC25A11 PE=1 SV=3</td></tr>
<tr><td>Q9NZ01</td><td>0.004506917</td><td>1</td><td>0</td><td>0</td><td>>sp|Q9NZ01|TECR_HUMAN Very-long-chain enoyl-CoA reductase OS=Homo sapiens GN=TECR PE=1 SV=1</td></tr>
<tr><td>Q9H082</td><td>0.007922216</td><td>1</td><td>0</td><td>0</td><td>>sp|Q9H082|RB33B_HUMAN Ras-related protein Rab-33B OS=Homo sapiens GN=RAB33B PE=1 SV=1</td></tr>
<tr><td>Q6PJF5</td><td>0.003818444</td><td>1</td><td>0.270247229</td><td>2</td><td>>sp|Q6PJF5|RHDF2_HUMAN Inactive rhomboid protein 2 OS=Homo sapiens GN=RHBDF2 PE=1 SV=2</td></tr>
<tr><td>O75396</td><td>0.001275752</td><td>1</td><td>0</td><td>0</td><td>>sp|O75396|SC22B_HUMAN Vesicle-trafficking protein SEC22b OS=Homo sapiens GN=SEC22B PE=1 SV=4</td></tr>
<tr><td>P61019</td><td>0.007333705</td><td>1</td><td>0</td><td>0</td><td>>sp|P61019|RAB2A_HUMAN Ras-related protein Rab-2A OS=Homo sapiens GN=RAB2A PE=1 SV=1</td></tr>
<tr><td>Q8TED1</td><td>0.002684051</td><td>1</td><td>0</td><td>0</td><td>>sp|Q8TED1|GPX8_HUMAN Probable glutathione peroxidase 8 OS=Homo sapiens GN=GPX8 PE=1 SV=2</td></tr>
<tr><td>P04406</td><td>0.003187823</td><td>1</td><td>0</td><td>0</td><td>>sp|P04406|G3P_HUMAN Glyceraldehyde-3-phosphate</td></tr>
</table>

					dehydrogenase OS=Homo sapiens GN=GAPDH PE=1 SV=3

Table S2

Pulldown proteins of ANKRD22 in organoid culture

Link	Pulldown molecules of ANKRD22		Pulldown molecules of negative control		Sequence Header
	Relative Abundance	Number of Peptides	Relative Abundance	Number of Peptides	
Q9BSJ8	17.05%	45	0	0	>sp|Q9BSJ8|ESYT1_HUMAN Extended synaptotagmin-1 OS=Homo sapiens GN=ESYT1 PE=1 SV=1
P07437	12.08%	16	0	0	>sp|P07437|TBB5_HUMAN Tubulin beta chain OS=Homo sapiens GN=TUBB PE=1 SV=2
P68363	14.08%	16	0	0	>sp|P68363|TBA1B_HUMAN Tubulin alpha-1B chain OS=Homo sapiens GN=TUBA1B PE=1 SV=1
P25705	1.75%	13	0	0	>sp|P25705|ATPA_HUMAN ATP synthase subunit alpha, mitochondrial OS=Homo sapiens GN=ATP5A1 PE=1 SV=1
P60709	10.73%	12	0	0	>sp|P60709|ACTB_HUMAN Actin, cytoplasmic 1 OS=Homo sapiens GN=ACTB PE=1 SV=1
P06576	2.72%	6	0	0	>sp|P06576|ATPB_HUMAN ATP synthase subunit beta, mitochondrial OS=Homo sapiens GN=ATP5B PE=1 SV=3
Q5VTE0	2.44%	5	0	0	>sp|Q5VTE0|EF1A3_HUMAN Putative elongation factor 1-alpha-like 3 OS=Homo sapiens GN=EEF1A1P5 PE=5 SV=1
P23396	1.49%	4	0	0	>sp|P23396|RS3_HUMAN 40S ribosomal protein S3 OS=Homo sapiens GN=RPS3 PE=1 SV=2
Q9H293	23.16%	4	0	0	>sp|Q9H293|IL25_HUMAN Interleukin-25 OS=Homo sapiens GN=IL25 PE=1 SV=1
P05141	3.26%	4	0	0	>sp|P05141|ADT2_HUMAN

					ADP/ATP translocase 2 OS=Homo sapiens GN=SLC25A5 PE=1 SV=7
P12235	1.78%	3	0	0	>sp|P12235|ADT1_HUMAN ADP/ATP translocase 1 OS=Homo sapiens GN=SLC25A4 PE=1 SV=4
P04792	0.71%	3	0	0	>sp|P04792|HSPB1_HUMAN Heat shock protein beta-1 OS=Homo sapiens GN=HSPB1 PE=1 SV=2
Q8NCF7	1.22%	3	0	0	>tr|Q8NCF7|Q8NCF7_HUMAN cDNA FLJ90278 fis, clone NT2RP1000325, highly similar to Phosphate carrier protein, mitochondrialprecursor OS=Homo sapiens PE=2 SV=1
Q5VW36	0.40%	3	0	0	>sp|Q5VW36|FOCAD_HUMAN Focadhesin OS=Homo sapiens GN=FOCAD PE=1 SV=1
P11142	0.33%	3	0	0	>sp|P11142|HSP7C_HUMAN Heat shock cognate 71 kDa protein OS=Homo sapiens GN=HSPA8 PE=1 SV=1
O15397	0.12%	3	0	0	>sp|O15397|IPO8_HUMAN Importin-8 OS=Homo sapiens GN=IPO8 PE=1 SV=2
P35579	0.02%	2	0	0	>sp|P35579|MYH9_HUMAN Myosin-9 OS=Homo sapiens GN=MYH9 PE=1 SV=4
Q92616	0.01%	2	0	0	>sp|Q92616|GCN1_HUMAN eIF-2-alpha kinase activator GCN1 OS=Homo sapiens GN=GCN1 PE=1 SV=6
Q9Y5L0	0.04%	2	0	0	>sp|Q9Y5L0|TNPO3_HUMAN Transportin-3 OS=Homo sapiens GN=TNPO3 PE=1 SV=3
A8K401	0.89%	2	0	0	>tr|A8K401|A8K401_HUMAN Prohibitin, isoform CRA_a OS=Homo sapiens GN=PHB PE=2 SV=1
P51159	2.25%	2	0	0	>sp|P51159|RB27A_HUMAN Ras-related protein Rab-27A OS=Homo sapiens GN=RAB27A PE=1 SV=3
Q9H0U4	0.34%	1	0	0	>sp|Q9H0U4|RAB1B_HUMAN Ras-related protein Rab-1B

					OS=Homo sapiens GN=RAB1B PE=1 SV=1
F8WE04	0.45%	1	0	0	>tr|F8WE04|F8WE04_HUMAN Heat shock protein beta-1 OS=Homo sapiens GN=HSPB1 PE=1 SV=1
Q9UNL2	0.29%	1	0	0	>sp|Q9UNL2|SSRG_HUMAN Translocon-associated protein subunit gamma OS=Homo sapiens GN=SSR3 PE=1 SV=1
P62917	0.16%	1	0	0	>sp|P62917|RL8_HUMAN 60S ribosomal protein L8 OS=Homo sapiens GN=RPL8 PE=1 SV=2
P09493	0.15%	1	0	0	>sp|P09493|TPM1_HUMAN Tropomyosin alpha-1 chain OS=Homo sapiens GN=TPM1 PE=1 SV=2
P43307	0.12%	1	0	0	>sp|P43307|SSRA_HUMAN Translocon-associated protein subunit alpha OS=Homo sapiens GN=SSR1 PE=1 SV=3
Q15800	0.12%	1	0	0	>sp|Q15800|MSMO1_HUMAN Methylsterol monooxygenase 1 OS=Homo sapiens GN=MSMO1 PE=1 SV=1
Q53GQ0	0.61%	1	0	0	>sp|Q53GQ0|DHB12_HUMAN Very-long-chain 3-oxoacyl-CoA reductase OS=Homo sapiens GN=HSD17B12 PE=1 SV=2
P31943	0.14%	1	0	0	>sp|P31943|HNRH1_HUMAN Heterogeneous nuclear ribonucleoprotein H OS=Homo sapiens GN=HNRNPH1 PE=1 SV=4
P81605	0.31%	1	0	0	>sp|P81605|DCD_HUMAN Dermcidin OS=Homo sapiens GN=DCD PE=1 SV=2
O14980	0.01%	1	0	0	>sp|O14980|XPO1_HUMAN Exportin-1 OS=Homo sapiens GN=XPO1 PE=1 SV=1
Q13535	0.06%	1	0	0	>sp|Q13535|ATR_HUMAN Serine/threonine-protein kinase ATR OS=Homo sapiens GN=ATR PE=1 SV=3
P39656	0.03%	1	0	0	>sp|P39656|OST48_HUMAN Dolichyl-diphosphooligosaccharide--

					protein glycosyltransferase 48 kDa subunit OS=Homo sapiens GN=DDOST PE=1 SV=4
P05091	0.07%	1	0	0	>sp|P05091|ALDH2_HUMAN Aldehyde dehydrogenase, mitochondrial OS=Homo sapiens GN=ALDH2 PE=1 SV=2
Q8IYD1	0.27%	1	0	0	>sp|Q8IYD1|ERF3B_HUMAN Eukaryotic peptide chain release factor GTP-binding subunit ERF3B OS=Homo sapiens GN=GSPT2 PE=1 SV=2
P0DMV9	0.01%	1	0	0	>sp|P0DMV9|HS71B_HUMAN Heat shock 70 kDa protein 1B OS=Homo sapiens GN=HSPA1B PE=1 SV=1
Q16891	0.04%	1	0	0	>sp|Q16891|MIC60_HUMAN MICOS complex subunit MIC60 OS=Homo sapiens GN=IMMT PE=1 SV=1
Q13200	0.01%	1	0	0	>sp|Q13200|PSMD2_HUMAN 26S proteasome non-ATPase regulatory subunit 2 OS=Homo sapiens GN=PSMD2 PE=1 SV=3
Q9P035	0.27%	1	0	0	>sp|Q9P035|HACD3_HUMAN Very-long-chain (3R)-3-hydroxyacyl-CoA dehydratase 3 OS=Homo sapiens GN=HACD3 PE=1 SV=2

Table S3

Pulldown proteins of nuclear ANKRD22 in organoid culture

Link	Number of peptides			Sequence Header
	Pulldown molecules of ANKRD22 in SW620	Pulldown molecules of ANKRD22 in RKO	Pulldown molecules of ANKRD22 in negative control	
Q15149	3	0	0	>sp|Q15149|PLEC_HUMAN Plectin OS=Homo sapiens GN=PLEC PE=1 SV=3
Q9UFH2	3	0	0	>sp|Q9UFH2|DYH17_HUMAN Dynein heavy chain 17, axonemal OS=Homo sapiens GN=DNAH17 PE=1 SV=2
P78527	6	0	0	>sp|P78527|PRKDC_HUMAN DNA-dependent protein kinase catalytic subunit OS=Homo sapiens GN=PRKDC PE=1 SV=3
P15924	4	0	0	>sp|P15924|DESP_HUMAN Desmoplakin OS=Homo sapiens GN=DSP PE=1 SV=3
Q13813	62	0	0	>sp|Q13813|SPTN1_HUMAN Spectrin alpha chain, non-erythrocytic 1 OS=Homo sapiens GN=SPTAN1 PE=1 SV=3
O15020	33	0	0	>sp|O15020|SPTN2_HUMAN Spectrin beta chain, non-erythrocytic 2 OS=Homo sapiens GN=SPTBN2 PE=1 SV=3
Q01082	17	0	0	>sp|Q01082|SPTB2_HUMAN Spectrin beta chain, non-erythrocytic 1 OS=Homo sapiens GN=SPTBN1 PE=1 SV=2
Q13085	13	0	0	>sp|Q13085|ACACA_HUMAN Acetyl-CoA carboxylase 1 OS=Homo sapiens GN=ACACA PE=1 SV=2
Q6P2Q9	2	0	0	>sp|Q6P2Q9|PRP8_HUMAN Pre-mRNA-processing-splicing

				factor 8 OS=Homo sapiens GN=PRPF8 PE=1 SV=2
A0A0U4BW16	268	0	0	>tr|A0A0U4BW16|A0A0U4BW16_HUMAN Non-muscle myosin heavy chain 9 OS=Homo sapiens GN=MYH9 PE=2 SV=1
P35579	306	1	0	>sp|P35579|MYH9_HUMAN Myosin-9 OS=Homo sapiens GN=MYH9 PE=1 SV=4
P13533	3	0	4	>sp|P13533|MYH6_HUMAN Myosin-6 OS=Homo sapiens GN=MYH6 PE=1 SV=5
Q86UU0	3	0	1	>sp|Q86UU0|BCL9L_HUMAN B-cell CLL/lymphoma 9-like protein OS=Homo sapiens GN=BCL9L PE=1 SV=1
Q9BZF9	2	0	0	>sp|Q9BZF9|UACA_HUMAN Uveal autoantigen with coiled-coil domains and ankyrin repeats OS=Homo sapiens GN=UACA PE=1 SV=2
Q9UM54	4	0	0	>sp|Q9UM54|MYO6_HUMAN Unconventional myosin-VI OS=Homo sapiens GN=MYO6 PE=1 SV=4
A6NGG8	1	0	0	>sp|A6NGG8|CB071_HUMAN Uncharacterized protein C2orf71 OS=Homo sapiens GN=C2orf71 PE=1 SV=1
Q12770	1	0	0	>sp|Q12770|SCAP_HUMAN Sterol regulatory element-binding protein cleavage-activating protein OS=Homo sapiens GN=SCAP PE=1 SV=4
Q08211	38	0	0	>sp|Q08211|DHX9_HUMAN ATP-dependent RNA helicase A OS=Homo sapiens GN=DHX9 PE=1 SV=4
Q13045	3	0	0	>sp|Q13045|FLII_HUMAN Protein flightless-1 homolog OS=Homo sapiens GN=FLII PE=1 SV=2
Q86SQ0	3	0	0	>sp|Q86SQ0|PHLB2_HUMAN Pleckstrin homology-like domain family B member 2 OS=Homo sapiens GN=PHLDB2 PE=1 SV=2

Q27J81	1	0	0	>sp|Q27J81|INF2_HUMAN Inverted formin-2 OS=Homo sapiens GN=INF2 PE=1 SV=2
P55265	2	0	0	>sp|P55265|DSRAD_HUMAN Double-stranded RNA-specific adenosine deaminase OS=Homo sapiens GN=ADAR PE=1 SV=4
A0A0D9SF60	2	0	0	>tr|A0A0D9SF60|A0A0D9SF60_HUMAN Plakophilin-4 OS=Homo sapiens GN=PKP4 PE=1 SV=1
Q9P2M7	2	0	0	>sp|Q9P2M7|CING_HUMAN Cingulin OS=Homo sapiens GN=CGN PE=1 SV=2
P11498	1	0	0	>sp|P11498|PYC_HUMAN Pyruvate carboxylase, mitochondrial OS=Homo sapiens GN=PC PE=1 SV=2
O00159	16	0	0	>sp|O00159|MYO1C_HUMAN Unconventional myosin-Ic OS=Homo sapiens GN=MYO1C PE=1 SV=4
Q7L014	1	0	0	>sp|Q7L014|DDX46_HUMAN Probable ATP-dependent RNA helicase DDX46 OS=Homo sapiens GN=DDX46 PE=1 SV=2
O14974	3	0	0	>sp|O14974|MYPT1_HUMAN Protein phosphatase 1 regulatory subunit 12A OS=Homo sapiens GN=PPP1R12A PE=1 SV=1
Q9H2U1	2	0	0	>sp|Q9H2U1|DHX36_HUMAN ATP-dependent RNA helicase DHX36 OS=Homo sapiens GN=DHX36 PE=1 SV=2
O94832	4	0	0	>sp|O94832|MYO1D_HUMAN Unconventional myosin-Id OS=Homo sapiens GN=MYO1D PE=1 SV=2
Q9Y2W1	4	0	0	>sp|Q9Y2W1|TR150_HUMAN Thyroid hormone receptor-associated protein 3 OS=Homo sapiens GN=THRAP3 PE=1 SV=2
Q9NYF8	4	0	0	>sp|Q9NYF8|BCLF1_HUMAN Bcl-2-associated transcription factor 1 OS=Homo sapiens GN=BCLAF1

				PE=1 SV=2
Q08043	1	0	0	>sp|Q08043|ACTN3_HUMAN Alpha-actinin-3 OS=Homo sapiens GN=ACTN3 PE=1 SV=2
P43243	1	0	0	>sp|P43243|MATR3_HUMAN Matrin-3 OS=Homo sapiens GN=MATR3 PE=1 SV=2
Q00839	5	0	0	>sp|Q00839|HNRPU_HUMAN Heterogeneous nuclear ribonucleoprotein U OS=Homo sapiens GN=HNRNPU PE=1 SV=6
P60709	32	10	5	>sp|P60709|ACTB_HUMAN Actin, cytoplasmic 1 OS=Homo sapiens GN=ACTB PE=1 SV=1
Q4KMQ1	1	0	0	>sp|Q4KMQ1|TPRN_HUMAN Taperin OS=Homo sapiens GN=TPRN PE=1 SV=2
Q14444	11	0	0	>sp|Q14444|CAPR1_HUMAN Caprin-1 OS=Homo sapiens GN=CAPRIN1 PE=1 SV=2
Q86VG2	3	0	0	>tr|Q86VG2|Q86VG2_HUMAN Splicing factor proline/glutamine-rich (Polypyrimidine tract binding protein associated) OS=Homo sapiens GN=SFPQ PE=2 SV=1
O95447	3	0	0	>sp|O95447|LCA5L_HUMAN Lebercilin-like protein OS=Homo sapiens GN=LCA5L PE=1 SV=1
Q16643	2	0	0	>sp|Q16643|DREB_HUMAN Drebrin OS=Homo sapiens GN=DBN1 PE=1 SV=4
Q9Y2M5	3	0	0	>sp|Q9Y2M5|KLH20_HUMAN Kelch-like protein 20 OS=Homo sapiens GN=KLHL20 PE=1 SV=4
B7Z344	1	0	0	>tr|B7Z344|B7Z344_HUMAN cDNA FLJ59625, highly similar to Rho guanine nucleotide exchange factor 7 OS=Homo sapiens PE=2 SV=1
Q59EI8	2	0	0	>tr|Q59EI8|Q59EI8_HUMAN Dual specificity phosphatase 5 variant (Fragment) OS=Homo sapiens PE=2 SV=1
Q99419	1	0	0	>tr|Q99419|Q99419_HUMAN

				ICSAT transcription factor (Fragment) OS=Homo sapiens PE=2 SV=1
A8K617	1	0	0	>tr|A8K617|A8K617_HUMAN cDNA FLJ78611 OS=Homo sapiens PE=2 SV=1
Q6RFH5	1	0	0	>sp|Q6RFH5|WDR74_HUMAN WD repeat-containing protein 74 OS=Homo sapiens GN=WDR74 PE=1 SV=1
Q6PJV4	3	0	0	>tr|Q6PJV4|Q6PJV4_HUMAN THRAP3 protein (Fragment) OS=Homo sapiens GN=THRAP3 PE=2 SV=1
Q96HJ3	3	0	0	>sp|Q96HJ3|CCD34_HUMAN Coiled-coil domain-containing protein 34 OS=Homo sapiens GN=CCDC34 PE=1 SV=2
Q9H293	3	0	6	>sp|Q9H293|IL25_HUMAN Interleukin-25 OS=Homo sapiens GN=IL25 PE=1 SV=1
Q562R4	2	1	1	>tr|Q562R4|Q562R4_HUMAN Actin-like protein (Fragment) OS=Homo sapiens GN=ACT PE=4 SV=1
I3L1Z6	2	0	0	>tr|I3L1Z6|I3L1Z6_HUMAN Multidrug resistance-associated protein 6 OS=Homo sapiens GN=ABCC6 PE=4 SV=1
A0N4V7	15	0	0	>tr|A0N4V7|A0N4V7_HUMAN HCG2039797 (Fragment) OS=Homo sapiens GN=Tcr-alpha PE=4 SV=1
Q8TF72	0	0	2	>sp|Q8TF72|SHRM3_HUMAN Protein Shroom3 OS=Homo sapiens GN=SHROOM3 PE=1 SV=2
Q9H0J4	0	0	2	>sp|Q9H0J4|QRIC2_HUMAN Glutamine-rich protein 2 OS=Homo sapiens GN=QRICH2 PE=1 SV=1
Q15058	0	0	2	>sp|Q15058|KIF14_HUMAN Kinesin-like protein KIF14 OS=Homo sapiens GN=KIF14 PE=1 SV=1
O75037	0	0	2	>sp|O75037|KI21B_HUMAN Kinesin-like protein KIF21B

				OS=Homo sapiens GN=KIF21B PE=1 SV=2
Q5SXM2	0	0	1	>sp|Q5SXM2|SNPC4_HUMAN snRNA-activating protein complex subunit 4 OS=Homo sapiens GN=SNAPC4 PE=1 SV=1
Q99490	0	0	2	>sp|Q99490|AGAP2_HUMAN Arf-GAP with GTPase, ANK repeat and PH domain-containing protein 2 OS=Homo sapiens GN=AGAP2 PE=1 SV=2
Q9NQW1	0	0	2	>sp|Q9NQW1|SC31B_HUMAN Protein transport protein Sec31B OS=Homo sapiens GN=SEC31B PE=1 SV=1
Q4VCS5	0	0	2	>sp|Q4VCS5|AMOT_HUMAN Angiomotin OS=Homo sapiens GN=AMOT PE=1 SV=1
O15083	0	0	4	>sp|O15083|ERC2_HUMAN ERC protein 2 OS=Homo sapiens GN=ERC2 PE=1 SV=3
O95503	0	0	2	>sp|O95503|CBX6_HUMAN Chromobox protein homolog 6 OS=Homo sapiens GN=CBX6 PE=1 SV=1
Q9UJZ1	0	0	2	>sp|Q9UJZ1|STML2_HUMAN Stomatin-like protein 2, mitochondrial OS=Homo sapiens GN=STOML2 PE=1 SV=1
U3KPU4	0	0	1	>tr|U3KPU4|U3KPU4_HUMAN GRIP and coiled-coil domain-containing protein 2 (Fragment) OS=Homo sapiens GN=GCC2 PE=1 SV=1
Q71U36	0	1	0	>sp|Q71U36|TBA1A_HUMAN Tubulin alpha-1A chain OS=Homo sapiens GN=TUBA1A PE=1 SV=1
P31943	0	1	0	>sp|P31943|HNRH1_HUMAN Heterogeneous nuclear ribonucleoprotein H OS=Homo sapiens GN=HNRNPH1 PE=1 SV=4
Q13885	0	1	0	>sp|Q13885|TBB2A_HUMAN Tubulin beta-2A chain OS=Homo sapiens GN=TUBB2A PE=1 SV=1

Q9HD23	0	1	0	>sp|Q9HD23|MRS2_HUMAN Magnesium transporter MRS2 homolog, mitochondrial OS=Homo sapiens GN=MRS2 PE=1 SV=1
Q9P035	0	1	0	>sp|Q9P035|HACD3_HUMAN Very-long-chain (3R)-3-hydroxyacyl-CoA dehydratase 3 OS=Homo sapiens GN=HACD3 PE=1 SV=2
P63244	0	1	0	>sp|P63244|RACK1_HUMAN Receptor of activated protein C kinase 1 OS=Homo sapiens GN=RACK1 PE=1 SV=3
Q99623	0	2	0	>sp|Q99623|PHB2_HUMAN Prohibitin-2 OS=Homo sapiens GN=PHB2 PE=1 SV=2
P09493	0	1	0	>sp|P09493|TPM1_HUMAN Tropomyosin alpha-1 chain OS=Homo sapiens GN=TPM1 PE=1 SV=2
P35232	0	3	0	>sp|P35232|PHB_HUMAN Prohibitin OS=Homo sapiens GN=PHB PE=1 SV=1

Table S4

Primers and probes used in RT-qPCR

Name	Sequence	Strand	Modification
ACC1	5'-TTCCGCAGAAAGGATCTGGT-3'	forward	
	5'-CAACTCCTTCCGCTCAGTTG-3'	reverse	
	5'-ACCATGCGTCGGGTGGACCCA-3'	Probe	5'Fam - 3'Tamra
FASN	5'-ACAGTGTCCTTGGAGGTACG-3'	forward	
	5'-TGGTACACCTTCCCACTCAC-3'	reverse	
	5'-CCTGGAGGCCACCGGTGCCT-3'	Probe	5'Fam - 3'Tamra
ACLY	5'-CTGATGCCATGAGGAAGCAC-3'	forward	
	5'-TCAGCTATGATGGCGATGGT-3'	reverse	
	5'-TGCCTCTCTCCGCTCTGCCT-3'	Probe	5'Fam - 3'Tamra
BBOX1	5'-ATGGCCGACGTAGCTATGAA-3'	forward	
	5'-GCCTTGACATGACCACATCC-3'	reverse	
	5'-CCCGCCATCTAGAAGGAGCTTATGCT-3'	Probe	5'Fam - 3'Tamra
PGC1A	5'-TGACTTTGACCCTGCTTCCA-3'	forward	
	5'-AGGTATTCGCCATCCCTCTG-3'	reverse	
	5'-ACCTGCGCAAGCTTCTCTGAGC-3'	Probe	5'Fam - 3'Tamra
CPT1A	5'-TGGACCGGGAGGAAATCAAA-3'	forward	
	5'-CATGTGCTGGATGGTGTCTG-3'	reverse	
	5'-CCGCTCCCACTGAGCGGAGCA-3'	Probe	5'Fam - 3'Tamra
ANKRD22	5'- CCAGCTTGGACTTCTAGGGA-3'	forward	
	5'-GGCAGATGGGCTCAGAGTAT-3'	reverse	
	5'-TCCCATGCTGGTCCTTCACAGG-3'	Probe	5'Fam - 3'Tamra
TUBB	5'-AGGAGGTCGATGAGCAGATG-3'	forward	
	5'-TTGCCAATGAAGGTGACTGC-3'	reverse	
	5'-TGTGACATCCCACCTCGTGGCC-3'	Probe	5'Fam - 3'Tamra
